# SnRK2 protein kinases represent an ancient system in plants for adaptation to a terrestrial environment

**DOI:** 10.1038/s42003-019-0281-1

**Published:** 2019-01-21

**Authors:** Akihisa Shinozawa, Ryoko Otake, Daisuke Takezawa, Taishi Umezawa, Kenji Komatsu, Keisuke Tanaka, Anna Amagai, Shinnosuke Ishikawa, Yurie Hara, Yasuko Kamisugi, Andrew C. Cuming, Koichi Hori, Hiroyuki Ohta, Fuminori Takahashi, Kazuo Shinozaki, Takahisa Hayashi, Teruaki Taji, Yoichi Sakata

**Affiliations:** 1 Department of Bioscience, Tokyo University of Agriculture, 1-1-1 Sakuragaoka, Setagayaku, Tokyo, 156-8502 Japan; 2 Graduate School of Science and Engineering, Saitama University, 255 Shimo-Okubo,Sakura-ku, Saitama, 338-8570 Japan; 3 Graduate School of Bio-Applications and Systems Engineering, Tokyo University of Agriculture and Technology, 2-24-16 Nakacho, Koganei, Tokyo, 184-8588 Japan; 4 Department of Bioproduction Technology, Junior College, Tokyo University of Agriculture, 1-1-1 Sakuragaoka, Setagayaku, Tokyo, 156-8502 Japan; 5 NODAI Genome Research Center (NGRC), Tokyo University of Agriculture, 1-1-1 Sakuragaoka, Setagayaku, Tokyo, 156-8502 Japan; 6 Centre for Plant Science, University of Leeds, Leeds, LS2 9JT UK; 7 School of Life Science and Technology, Tokyo Institute of Technology, 4259 Nagatsuta-cho, Midori-ku, Yokohama, Kanagawa 226-8503 Japan; 8 Gene Discovery Research Group, RIKEN Center for Sustainable Resource Science, 3-1-1, Koyadai, Tsukuba, Ibaraki, 305-0074 Japan

## Abstract

The SNF1-related protein kinase 2 (SnRK2) family includes key regulators of osmostress and abscisic acid (ABA) responses in angiosperms and can be classified into three subclasses. Subclass III SnRK2s act in the ABA response while ABA-nonresponsive subclass I SnRK2s are regulated through osmostress. Here we report that an ancient subclass III SnRK2-based signalling module including ABA and an upstream Raf-like kinase (ARK) exclusively protects the moss *Physcomitrella patens* from drought. Subclass III SnRK2s from both Arabidopsis and from the semiterrestrial alga *Klebsormidium nitens*, which contains all the components of ABA signalling except ABA receptors, complement *Physcomitrella snrk2*^−^ mutants, whereas Arabidopsis subclass I SnRK2 cannot. We propose that the earliest land plants developed the ABA/ARK/subclass III SnRK2 signalling module by recruiting ABA to regulate a pre-existing dehydration response and that subsequently a novel subclass I SnRK2 system evolved in vascular plants conferring osmostress protection independently from the ancient system.

## Abstract (web summary)

Akihisa Shinozawa, Ryoko Otake et al. report on an ancient subclass of the SNF1-related protein kinase 2 family involved in ABA signaling, which protect moss from drought stress. They propose that this signaling module evolved in early land plants to regulate dehydration-response.

## Introduction

Due to their sessile nature, land plants have developed various strategies to cope with dehydration under water-deficit conditions. In addition to anatomical adaptations such as development of roots, vascular systems and cuticle-coated epidermal layers with stomata for efficient water usage, land plants also developed signal transduction systems for sensing and responding to water-deficient conditions. Osmostress-responsive accumulation of the phytohormone ABA triggers responses such as stomatal closure, and accumulation of late embryogenesis abundant (LEA) proteins and soluble sugars that protect cellular functions from dehydration^1^. ABA signalling acts through protein phosphorylation via SnRK2 kinases^2–5^. These are classified in three subclasses (I–III)^6^, and the subclass III SnRK2s are an essential component for ABA signal transduction regulated by soluble ABA receptors (PYR/PYL/RCAR) and Group A protein phosphatase 2Cs (PP2CAs) in angiosperms^7,8^. PP2CAs of Arabidopsis negatively regulate ABA signalling by inhibiting subclass III SnRK2 activity through direct interaction. The inhibition is cancelled when ABA binds to PYR/PYL/RCAR, which specifically sequester the PP2CAs, facilitating SnRK2 activation^2,9,10^. SnRK2s are also activated by osmostress. Disruption of all three subclasses in Arabidopsis results in a loss of osmostress responses^11^. However, it is yet to be elucidated how ABA-nonresponsive SnRK2 (subclass I) is regulated through osmostress.

Although SnRK2s are evolutionarily conserved among land plants^12^, our knowledge of SnRK2 functions in non-angiosperms is limited. The genome of the model moss *Physcomitrella patens* encodes only four *SnRK2* genes (*PpSnRK2A* / *2B* / *2C* / *2D*), all of which belong to subclass III^13^. *PpSnRK2A/ Pp OPEN STOMATA1 (OST1)* restores ABA responsiveness of stomatal closure in the Arabidopsis *snrk2.6/ost1* mutant, and disruption of *PpSnRK2A/PpOST1* results in defective ABA-responsive closure of moss stomata, which occur only at the base of sporophytes, the non-dominant generation in bryophytes^14^. However, it is not known whether the *Ppsnrk2a/Ppost1* plant also has a defect in the ABA responsiveness of the moss protonemata, where ABA responses as well as tolerance to desiccation and osmostress have been well documented^15–19^.

Our previous studies have highlighted the differential regulation of ABA signalling between *P. patens* and Arabidopsis. We have shown that PP2CAs of *P. patens* play a major role in repressing ABA signalling downstream of the 40 kDa ABA-activated kinases, suggesting a novel mechanism to regulate the kinases^20^. In addition, we identified a novel ABA-activated Raf-like kinase (ARK), also known as ABA NON-RESPONSIVE^21^ or CONSTITUTIVE TRIPLE RESPONSE1-LIKE^22^, that acts upstream of the 40 kDa ABA-activated kinases and is essential for ABA responses in *P. patens*^21,23^. In this study, to understand the function of SnRK2s in bryophytes, we establish *Ppsnrk2* null mutants of *P. patens* using sequential gene targeting. We show that a high level of osmostress tolerance in *P. patens* is achieved exclusively through an ABA–SnRK2 signalling module. The highly conserved motif in the activation loop of land plant subclass III SnRK2s, which is the phosphorylation target of ARK^23^, plays a crucial role in SnRK2 activation. A cross-species complementation assay demonstrates that a *SnRK2* gene from the semiterrestrial alga *Klebsormidium nitens*, which contains all the components of ABA signalling except ABA receptors^24^ can complement *Ppsnrk2* mutants. We propose that the ABA/ARK/subclass III SnRK2 signalling module is an ancestral feature that enabled the colonization of land by simple plants, which subsequently recruited ABA to modulate the dehydration response before the separation of bryophytes and vascular plants. Subsequently, vascular plants further developed a novel subclass I SnRK2 signalling module for osmostress tolerance independent of the ABA-ARK system.

## Results

### Role of SnRK2 in ABA- and osmostress responses of *P. patens*

We established *Ppsnrk2* null mutants of *P. patens* using sequential gene targeting of the *Ppsnrk2a (Ppost1*) plant (SKO)^14^ to generate the *Ppsnrk2a/b* double knockout (DKO), the *Ppsnrk2a/b/c* triple knockout (TKO), and two independent *Ppsnrk2a/b/c/d* quadruple knockouts (QKOs; line #3 and #7) (Supplementary Fig. 1a). The disruption of the four *PpSnRK2* genes through homologous recombination in QKO plants was confirmed by PCR (Supplementary Figs. 1b and 1c) and Southern blot analysis (Supplementary Fig. 1d). Null expression of *PpSnRK2* genes in QKO (line #7) was confirmed by RNA-seq analysis (Supplementary Fig. 1e).

**Fig. 1:**
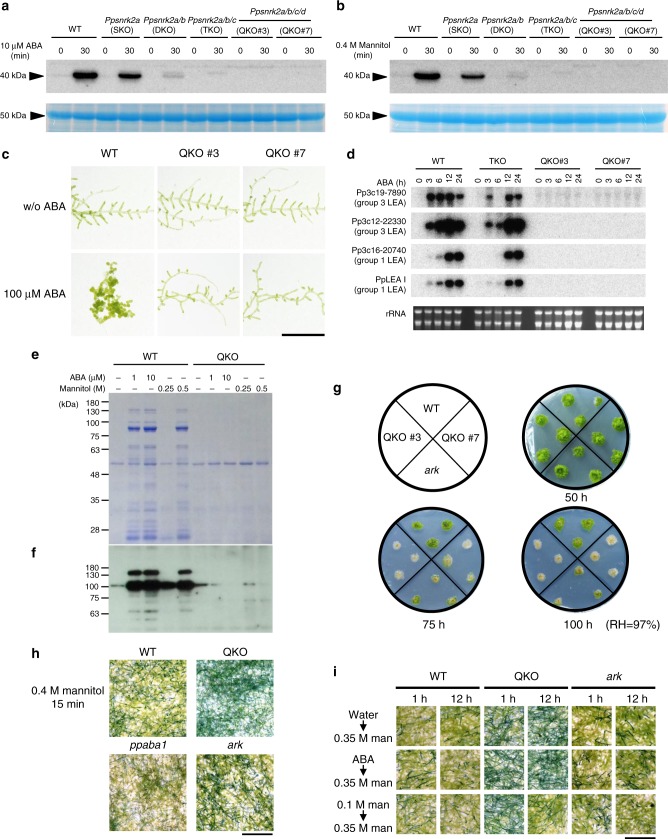
Essential role of PpSnRK2 in ABA responses and osmostress tolerance. **a**, **b** Activity of kinases from ABA-treated (**a**) or osmostress-treated (**b**) protonemata of wild-type (WT) and *Ppsnrk2* disruptants. Total soluble proteins extracted from protonemata treated with 10 µM ABA or with 0.4 M mannitol for indicated periods were subjected to in-gel phosphorylation assay using histone IIIS as a substrate. The bottom panel shows Coomassie Brilliant Blue (CBB) staining of the protein samples to show equal loading. **c** Protonemal growth of WT and the *Ppsnrk2* QKO plants with or without 100 µM ABA. **d** RNA gel blot analysis of ABA-inducible *LEA* gene expressions of WT, *Ppsnrk2* TKO, and QKO. Protonemata were treated with 10 µM ABA for indicated periods, and total RNA was used for the analysis. The bottom panel shows ethidium bromide staining of rRNA to show equal loading. **e**, **f** Accumulation of **e** boiling soluble proteins and **f** a LEA (17B9) protein in protonemata of WT or QKO plants treated with ABA or mannitol. Protonemata were treated with or without the indicated concentration of ABA or mannitol for 1 d prior to protein extraction. **e** Extracted proteins were boiled, and the supernatant (boiling soluble proteins) was separated by SDS-PAGE and stained by CBB. **f** Extracted proteins were separated by SDS-PAGE, transferred onto a nylon membrane, and reacted with anti-17B9 antibody. **g** Desiccation tolerance of *Ppsnrk2* QKO plants. The ABA-insensitive mutant *ark* was used as a control. Protonemata were dehydrated in an atmosphere of relative humidity of 97% for indicated periods. The dehydrated tissues were hydrated in water and grown on normal medium for two weeks. **h** Comparison of osmoshock tolerance among ABA mutants of *P. patens*. Protonemata of WT, QKO, ABA-deficient *Ppaba1*, and *ark* were treated with 0.4 M mannitol solution for 15 min, then stained by 0.5% Evans Blue. **i** Protonemata of WT, QKO, and *ark* were subjected to an acclimation assay. One-week-old protonemata were treated with 0.1 M mannitol solution or 10 µM ABA for 1 h or 12 h, then subjected to osmoshock treatment with 0.35 M mannitol for 20 min and stained with 0.5% Evans Blue. Scale bars, 0.5 cm in **c**, **h**, **i**

Previously we identified a 40 kDa kinase from *P. patens* protonemata activated by ABA and osmostress^20,23^. To confirm the identity of the kinase activity, proteins extracted from ABA- or mannitol-treated protonemata of wild-type and *Ppsnrk2* disruptants were subjected to the in-gel kinase assay (Figs. 1a, b). A decrease of kinase activity correlated with the number of disrupted genes, QKO plants showing little kinase activity after treatments. These results strongly support identification of the 40 kDa kinases as PpSnRK2s. ABA responses of *P. patens* protonemata were also adversely affected in the *SnRK2* disruptants (Supplementary Fig. 2a). Growth of wild-type protonemata is effectively repressed by ABA treatment, with structural changes leading to the formation of thick-walled spherical brood cells^25,26^. Protonemal growth of wild-type and *Ppsnrk2* SKO, DKO, and TKO plants was inhibited on medium containing 10 µM ABA, but QKO plants showed little growth inhibition, similar to the response of the ABA-insensitive *ark* mutant, which lacks functional ARK^23^. Even with ABA concentration increased to 100 µM, few brood cells were observed in QKO plants **(**Fig. 1c**)***. P. patens* protonemata are susceptible to freezing temperatures, but ABA pre-treatment markedly enhances freezing tolerance^27^. All plants except QKO mutants showed ABA-induced freezing tolerance comparable with that of wild-type (Supplementary Fig. 2b). Exogenous ABA also induces desiccation tolerance of *P. patens* protonemata^17,18,28^, and we found that ABA-treated wild-type protonemata could survive desiccation in an atmosphere of 75% relative humidity (RH) (−39 MPa)^17^ for 24 h. By contrast, ABA-treated QKO plants and the *ark* mutant could not (Supplementary Fig. 2c).

Hydrophilic late embryogenesis abundant (LEA) proteins are associated with desiccation tolerance of plant cells^20,29^. We confirmed the lack of expression of several *LEA* genes in QKO plants in response to ABA by RNA gel blot analysis (Fig. 1d). We further investigated the accumulation of boiling-soluble proteins, which is a feature of hydrophilic LEA proteins^15^. Exogenous ABA and mannitol-induced osmostress caused accumulation of boiling-soluble proteins in wild-type, but not in QKO plants (Fig. 1e). Immunoblot analysis using a specific antibody to 17B9 (Pp3c23_13700V3.1), a LEA-like protein associated with the osmostress response^30^ confirmed this (Fig. 1f). We then evaluated the osmostress tolerance of QKO plants. We dehydrated protonemal cells in an atmosphere of 97% RH (corresponding to −4 MPa water potential) for 50, 75, and 100 h (Fig. 1g). As previously reported, this treatment reduces water content of the protonemata to about 60 (50 h), 45 (75 h), and 40% (100 h), respectively^17^. These protonemata were rehydrated and then recovered on normal medium. Both QKO plants and the *ark* mutant survived dehydration for 50 h like wild-type plants, suggesting that PpSnRK2s and ARK are not essential for mild (60% water content) dehydration stress-tolerance. By contrast, whereas wild-type plants survived 75–100 h of dehydration, the QKO plants and the *ark* mutant did not. QKO plants were also hypersensitive to osmotic stress. Protonemata of QKO as well as the *ark* mutant could not survive after culture on medium containing 1 M mannitol (−2.5 MPa) (Supplementary Fig. 2d). Furthermore, when wild-type protonemata were subjected to osmoshock by exposure to a moderate concentration of 0.4 M mannitol solution for 15 min, the majority of cells survived, as judged by Evans blue staining (Fig. 1h), whereas most protonemal cells of the QKO plants did not survive this treatment. We also compared osmoshock tolerance of QKO with both ABA-deficient transgenic *P. patens* (*ppaba1*)^30^ and the *ark* mutant. The ABA-deficient mutant and to a lesser extent the *ark* mutant was tolerant to this treatment; however, QKO was not. This suggests that an additional ABA- and ARK- independent SnRK2 function also enables wild-type cells to withstand osmoshock. It is possible that even without osmostress PpSnRK2 might be slightly activated through unknown upstream kinase(s) or by autophosphorylation. Nevertheless, QKO protonemata were susceptible even to mild osmoshock imposed by 0.35 M mannitol; however, if pretreated with a non-detrimental osmostress (0.1 M mannitol) for 12 h, they acquired tolerance to 0.35 M mannitol (Fig. 1i). In contrast, ABA-pretreatment did not recover the osmotolerance of QKO protonemata, indicating that osmostress acclimation of *P. patens* involves an ABA- and SnRK2-independent system. Analysis of *Ppsnrk2a/b/d* TKO plants showed these to have ABA sensitivity comparable with wild-type (Supplementary Fig. 2e). Moreover, introduction of *PpSnRK2A* or -*D* sequences under the constitutive *actin* promoter was sufficient to complement the QKO mutant phenotype, restoring ABA sensitivity to protonemata (Supplementary Fig. 2f). This demonstrates that *PpSnRK2* genes are functionally redundant and play a major role in protonemal ABA signalling. Our data clearly demonstrated the essential role of SnRK2 in ABA signalling as well as in dehydration tolerance of the moss protonemata.

### SnRK2 in global gene expression and protein phosphorylation

**Fig. 2:**
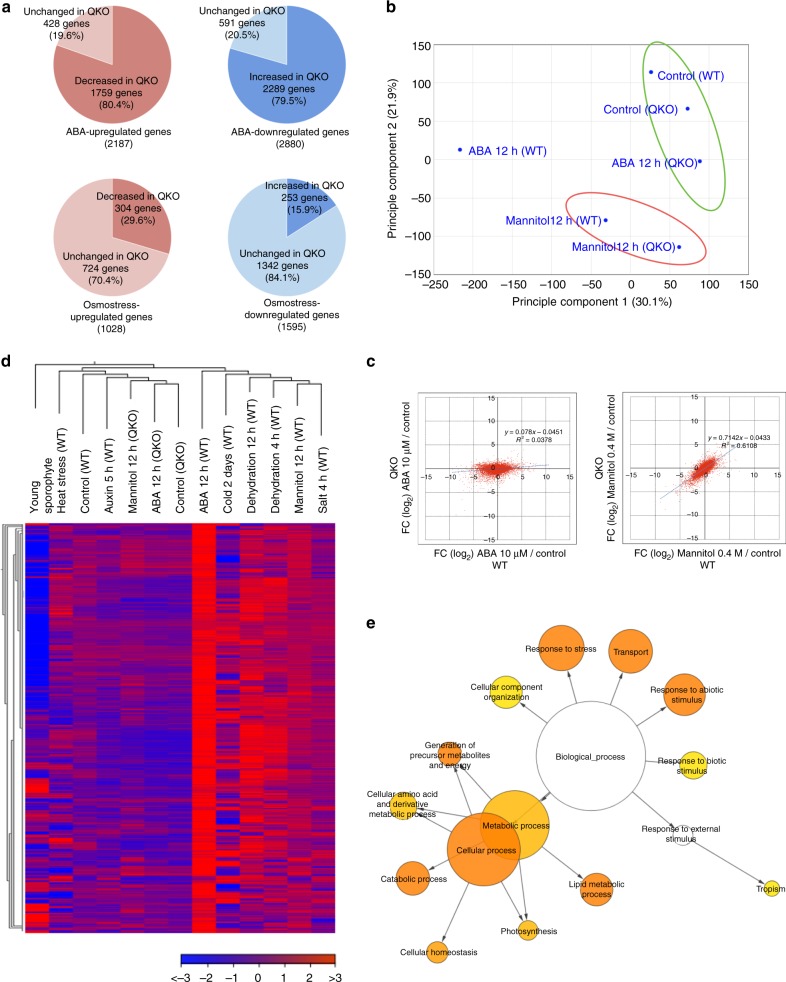
Transcriptomic analysis of PpSnRK2 downstream factors. **a** Numbers of ABA-regulated genes and osmostress-regulated genes in wild-type (WT), and the numbers of genes affected in QKO plants. Genes with more than two-fold difference in the expression level between ABA-treated WT and QKO or mannitol-treated WT and QKO (false discovery rate (FDR) ≤ 0.05) were categorized as Decreased or Increased in QKO. **b** Principal component analysis of gene expression profile in QKO plants. **c** Fold-changes (FC) (log2) after 12 h of (left) 10 µM ABA treatment or (right) 0.4 M mannitol treatment of whole genes detected in the RNA-seq experiment were plotted. X-axis, WT; y-axis, QKO. **d** Hierarchical clustering and heatmap analysis were performed for the SnRK2-regulated ABA-inducible genes (SAGs). Gene expression values are represented as relative to the mean of all samples; blue represents lower expression, red represents higher expression (expression range, −3 to 3). Following high-throughput sequencing, data were obtained from the NCBI Sequence Read Archive, Auxin 5 h (SRR3350456), Dehydration 4 h (SRR1707403), Dehydration 12 h (SRR2225592), Heat stress (SRR790663), Salt 4 h (SRR1707400), and Young sporophyte (SRR1553314). **e** Gene ontology term enrichment analysis was performed against SAGs. Functional enrichment analysis was performed for BLAST best-hit genes of SAGs, which were used to search Arabidopsis TAIR 10 proteins using the BiNGO plug-in in Cytoscape. Gene ontology Slim was used to summarize the network. The intensity of the color indicates significance of nodes based on corrected p-value (5% FDR)

To investigate the effect of *SnRK2* disruption on global gene expression we used RNA-seq analysis of wild-type and QKO plants treated with 10 µM ABA (12 h) or subjected to osmostress with 0.4 M mannitol (12 h) (Supplementary Data 1). The RNA-seq data were verified by qPCR analysis of the response of signature ABA/stress-response genes (Supplementary Fig. 3). In wild-type plants, 16,924 genes were represented by more than five mapped reads per transcript. Of these 2187 were significantly (>2 fold with a false discovery rate with *P* values less than 0.05) upregulated and 2880 were down-regulated by ABA treatment. Osmotic stress resulted in 1028 genes being significantly upregulated and 1595 down-regulated (Supplementary Fig. 4a). The majority of osmostress-responsive genes overlapped with ABA-responsive genes, suggesting that osmostress-induced ABA accumulation is responsible for these expression patterns. The number of ABA-responsive genes was greatly reduced in QKO plants whereas that of osmostress-responsive genes was relatively normal (Fig. 2a). Principal component analysis of gene expression profiles in QKO plants indicated that the effect of *PpSnRK2* disruption appeared most prominently in the ABA response (Fig. 2b), as was also indicated by scatter plots of gene expression of QKO vs wild-type after ABA treatment (*R*^2^ = 0.0378) compared to that of osmostress treatment (*R*^2^ = 0.6108) (Fig. 2c). We found a significant number of genes that were affected only by osmostress in wild-type (Supplementary Fig. 4a). Interestingly, expression of these genes was scarcely affected by disruption of *SnRK2*. This cohort of ABA- and SnRK2-independent osmostress responsive genes represent candidate components of the osmostress acclimation process. It is noteworthy that RNA-seq analysis also demonstrated that the expression of many genes, including *LEA* (Supplementary Fig. 4b), were more drastically reduced in the QKO mutant than in the wild-type, under normal conditions. This may partly explain the reason for the hypersensitivity of QKO plants following osmoshock (Fig. 1h).

To gain insights into the characteristics of SnRK2-regulated ABA-upregulated genes (SAGs), we performed hierarchical clustering and heatmap analysis using various public *P. patens* RNA-seq data^21,31–33^. The SAGs were also upregulated by typical dehydration-related stresses (Fig. 2d). Functional categorization of SAGs, by gene ontology term enrichment analysis revealed that SnRK2s are involved mainly in abiotic stress responses and energy metabolism (Fig. 2e). We compared our transcriptomic data with that of the *ark* mutant^23^ for ABA-responsive genes (Supplementary Fig. 4c). Most ARK-regulated genes (91% for ABA-induced and 77% for ABA-repressed) were included in SnRK2-regulated genes, indicating the major role of the ABA/ARK/SnRK2 module in the regulation of ABA-responsive gene expression. To investigate the overlap between responses of SnRK2-regulated gene expression in *P. patens* and Arabidopsis, we analysed transcriptomic data for the Arabidopsis *srk2dei* mutant, which lacks subclass III SnRK2, deposited in ArrayExpress (E-MEXP-3713;^34^) (Supplementary Fig. 4d and Supplementary Data 2). We found that about 20% of Arabidopsis subclass III-regulated ABA responsive genes were orthologues of *P. patens* SnRK2-regulated, ABA-responsive genes. Gene ontology term enrichment analysis of the overlapping gene sets showed enrichment of stress responsive genes (Supplementary Fig. 4e), indicating SnRK2 function to be evolutionarily conserved in regulating stress responses of land plants. We also investigated genes with fewer than 5 mapped reads in any samples, which were removed from analyses described above. These genes also contained a significant number of ABA- and SnRK2-regulated genes such as *LEA* genes (Supplementary Figure 5a and Supplementary Data 3). Gene ontology term enrichment analysis of these genes resulted in a similar pattern with that of SAGs (Supplementary Figure 5b).

**Fig. 3:**
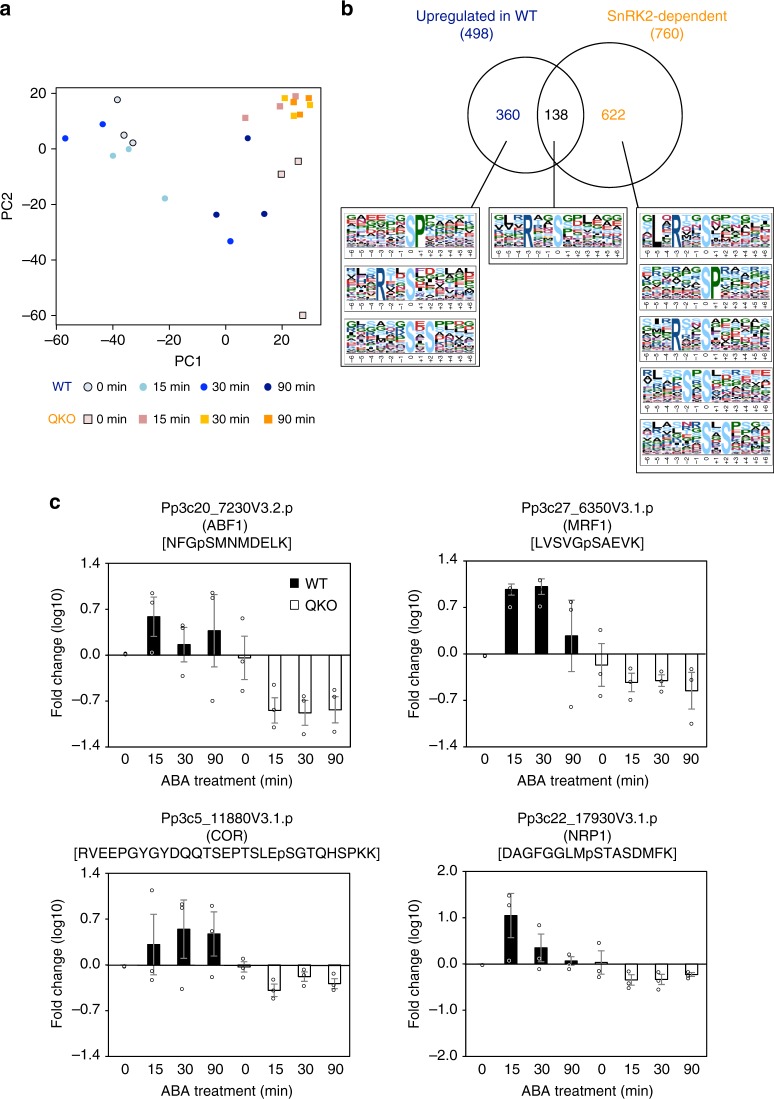
Phosphoproteomic analysis of *P. patens* WT and *Ppsnrk2* QKO plants. **a** Principal component analysis of quantitative data of phosphopeptides from wild-type (WT) and QKO plants under ABA treatment. **b** A Venn diagram of two phosphopeptide groups; one is the ABA-responsive (upregulated) group in WT, and the other is the SnRK2-dependent group. Phosphorylation motifs in those phosphopeptides were analyzed by the Motif-X algorithm. **c** Examples of ABA-responsive phosphopeptides in WT (solid) and QKO (empty). Pp3c20_7230V3.2.p; ABF transcription factor, Pp3c27_6350V3.1.p; MRF1 (MA3 domain-containing translation regulatory factor), Pp3c5_11880V3.1.p; COR (dehydrin), and Pp3c22_17930V3.1.p; NRP1 (nucleosome assembly protein 1). The relative peak areas to that of WT control (0 min) for respective phosphopeptides were calculated in each replicate using the quantitative data described in Supplementary Data 4. The values are mean ± S.E. (*n* = 3)

In addition to the RNA-seq analysis, we performed phosphoproteome analysis to investigate the effect of *SnRK2* deletion on ABA-responsive protein phosphorylation^34^. We detected 3789 unique phosphopeptides in protonemal proteins prepared from wild-type and QKO treated with or without ABA. Of these, 760 showed significant differences between the wild-type and QKO, and principal component analysis demonstrated that disruption of PpSnRK2 greatly affected the phosphoproteome profiles (Fig. 3a). In wild-type plants, 498 phosphopeptides showed a more than 3-fold change in abundance after ABA treatment in at least two of the three biological replicates. In QKO plants, 138 (27.8%) of the 498 ABA-responsive phosphopeptides (corresponding to 105 unique proteins) were lower in abundance (1/3-fold compared to wild-type) (Fig. 3b and Supplementary Data 4 and 5). Amagai et al. reported through the phosphoproteomic analysis of the *ark* mutant that ARK regulated 126 (88.1 %) of the 143 ABA-responsive phosphopeptides^35^. We compared the ARK-regulated ABA-responsive phosphopeptides with the 138 ABA-responsive phosphopeptides regulated by PpSnRK2. The result showed that 24 proteins were commonly regulated by ARK and SnRK2, which included an ABA-responsive bZIP transcription factor (ABF)-related protein (Pp3c20_7230V3.2.p). Examples of these ABA-responsive phosphorylation profiles between wild-type and QKO are shown (Fig. 3c). An in vitro phosphorylation assay was performed to confirm whether SnRK2 could phosphorylate selected phosphopeptides identified in this study. We chose 3 phosphopeptides from the list of PpSnRK2-regulated phosphopeptides and one phosphopeptide which is not included in the list, then these peptides were subjected to in vitro phosphorylation assay by PpSnRK2B (Supplementary Figure 6 and Supplementary Data 6). Our data demonstrated that two peptides derived from a kinesin motor protein and a serine/threonine-protein kinase as well as a positive control peptide derived from a sucrose-phosphate synthase, which was shown to be phosphorylated by PpSnRK2B in vitro^35^, were strongly phosphorylated by PpSnRK2B, confirming that our phosphoproteomic data involved SnRK2 substrates. On the other hand, a PpSnRK2-regulated phosphopeptide from phototropin was only weakly phosphorylated to a similar extent with that of a phosphopeptide not regulated by PpSnRK2 derived from a calmodulin-binding transcription activator (CAMTA), suggesting that the phototropin may not be directly phosphorylated by PpSnRK2. It has been demonstrated in Arabidopsis that ABFs are SnRK2 substrates^34,36,37^ and four ABF transcription factors (ABF1, ABF2, ABF3, and ABF4) are the master regulators for SnRK2-activated ABA-responsive transcription in vegetative tissues^38,39^. Our analysis in *P. patens* detected a conserved, ABA-responsive, SnRK2-dependent phosphopeptide NFGpSMNMDEFLK in a bZIP transcription factor, which is also found in the C1 domain of Arabidopsis ABFs and which is known to be phosphorylated by Arabidopsis subclass III SnRK2 in an ABA-dependent manner^34^. Our data suggest that ARK and SnRK2 together regulate ABA signalling through phosphorylation of ABFs in *P. patens* and that the core ABA phosphosignalling is evolutionarily conserved between bryophytes and angiosperms. The Motif-X algorithm detected four motifs; [–R–x–x–pS–], [–pS–P–], [–pS–x–S–] and [–S–x–pS] from the phosphopeptides enhanced in response to ABA (Fig. 3b and Supplementary Data 7). The motif [–R–x–x–pS–] was the only sequence common to both ABA-enhanced and SnRK2-dependent datasets. This motif was also identified as a phosphorylation target motif of Arabidopsis subclass III SnRK2^38,34^, representing the evolutionarily conserved phosphorylation preference in subclass III SnRK2 in land plants. We also detected the [–pS–P–] motif in SnRK2-independent ABA-responsive phosphopeptides. This motif is a minimal mitogen-activated protein kinase (MAPK) target motif, suggesting an involvement of MAPK cascade in ABA signalling of the moss, as demonstrated in Arabidopsis^34,39^. The [–pS/pT–x–S–] and [–S–x–pS/pT–] motifs were not detected in previous phosphoproteome analyses of Arabidopsis SnRK2 or of *Physcomitrella* ARK, suggesting that these motifs are species-specific recognition motifs of SnRK2 in the moss and represent differences in ABA-responsive phosphosignaling between bryophytes and angiosperms^35^. It is noteworthy that there were only three overlaps of SnRK2-regulated proteins between *P. patens* and Arabidopsis^34,39^ {AT3G13300/Pp3c15_3450V3.1.p (varicose); AT3G57150/Pp3c22_190V3.1.p (putative pseudouridine synthase NAP57); AT1G49720.1/ Pp3c20_7230V3.2.p (ABF1 abscisic acid responsive element-binding factor 1)}. This observation may imply the differential evolution of ABA-responsive phosphosignalling downstream of SnRK2 after the separation of bryophytes and vascular plants, as suggested in the previous report^35^. Nevertheless, 360 peptides were phosphorylated in response to ABA in QKO plants, suggesting the presence of an alternative, SnRK2-independent, phosphorylation cascade(s) in response to ABA.

### Activation of SnRK2 via the ARK recognition motif

**Fig. 4:**
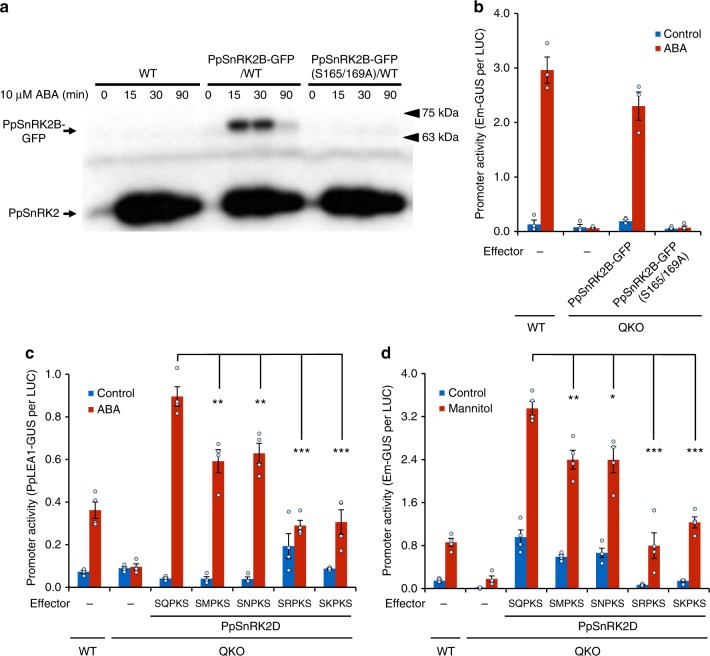
ABA-activated SnRK2 activity of *P. patens* depends on the evolutionarily conserved ARK phosphorylation motif in the activation loop. **a** Protonemata of wild-type (WT) or transgenic plants expressing SnRK2B-GFP or mutated SnRK2-GFP (S165/169 A) under the control of the rice *actin* promoter were treated with or without 10 µM ABA for the indicated periods in minutes, then the protein extracts were subjected to in-gel kinase assay. Arrows indicate the position of recombinant SnRK2B (SnRK2-GFP) and endogenous SnRK2. **b** Constitutive rice *actin* promoter–driven SnRK2B-GFP or mutated SnRK2-GFP (S165/169 A) in the predicted phosphorylation sites was introduced into QKO protonemata with *Em-GUS* and *Ubi-LUC* by particle bombardment. Protonemal cells were incubated with or without 10 µM ABA for 1 day, then subjected to GUS and LUC assays. Promoter activity of the *Em* promoter in WT and QKO in the absence of the effector construct is shown as controls. Levels of gene expression are represented by GUS per LUC ratio. Error bars indicate the standard error (SE; *n* = 4). **c**, **d** *PpSnRK2D* or its mutant variants fused to *PpSnRK2D* gene native promoter were introduced into QKO protonemata with *PpLEA1-GUS* for 10 µM ABA treatment for 1 d (**c**), or with *Em-GUS* for 0.4 M mannitol treatment for 1 d (**d**). Promoter activity of promoters in WT and QKO in the absence of the effector construct are shown as controls. Levels of gene expression are represented by the GUS per LUC ratio. Error bars indicate the SE (*n* = 4). *P < 0.05, **P < 0.01, ***P < 0.001 compared with the *PpSnRK2D* effector of QKO with ABA

Previously, we showed that the Raf-like kinase ARK plays an essential role in ABA signalling in moss and that the *ark* mutant failed to activate the ABA-activated kinases^23^, which are shown to be PpSnRK2s in this study. In vitro, ARK phosphorylated Ser-165 and/or Ser-169 (SQPKS) within the activation loop of PpSnRK2B that elicits the kinase activity^23^. We generated transgenic plants over-expressing green fluorescent protein (GFP) fusion proteins of PpSnRK2B (PpSnRK2B-GFP) or the mutated protein in which two serine residues were substituted for alanine residues (PpSnRK2B-GFP(S165/169 A)) in a wild-type background. The ABA-induced kinase activity of the wild-type PpSnRK2B-GFP protein was abolished by Ala substitutions of Ser-165 and Ser-169 (S165/169 A) (Fig. 4a). We also performed functional complementation by the PpSnRK2B protein by a transient assay. Transcription from a wheat *Em* promoter is activated by ABA in moss, and can be monitored by the transient assay of protonemal cells using particle bombardment of the *Em* promoter fused to β-glucuronidase (GUS) reporter gene (*Em-GUS*)^40^. ABA-responsive *Em-GUS* expression was abolished in the QKO protonemata (Fig. 4b). Co-introduction of the *PpSnRK2B-GFP* effector construct with *Em-GUS* into the QKO protonemata restored ABA-responsive *Em-GUS* expression; however, *PpSnRK2B-GFP (S165/169* *A)* completely failed to restore the ABA response. The core sequence SQPKS is evolutionarily conserved among subclass III SnRK2s, but it has been changed to S[K/R/N/M]PKS in subclass I SnRK2s (Supplementary Fig. 7a). To confirm the importance of the conserved SQPKS motif for the subclass III SnRK2 activation, we introduced mutations into PpSnRK2D to change the consensus sequence to the subclass I-type and monitored activity using an endogenous ABA-responsive *PpLEA1* promoter (*PpLEA1-GUS*)^41^ (Fig. 4c). The single mutation of Gln to either Met or Asn significantly reduced the activation of *PpLEA1-GUS*. Mutation to the basic amino acids Arg or Lys (the most frequent amino acids in the subclass I motif) further decreased PpSnRK2D effector activity. We also tested the activation of *Em-GUS* by osmostress because *PpLEA1-GUS* did not show osmostress-dependent activation in this assay (Supplementary Fig. 7b). The mutated versions of PpSnRK2D all showed reduced activity in response to osmostress, comparable with the reductions in ABA-responsiveness (Fig. 4d). We further confirmed the importance of the SQPKS motif in vitro. PpSnRK2D with or without mutations fused to maltose-binding protein (MBP) and the kinase domain of ARK fused to glutathione S-transferase (GST) were expressed in *Escherichia coli*, and affinity-purified proteins were subjected to in vitro phosphorylation assay. Although mutations of Gln to either Met or Asn did not show any significant effect on phosphorylation by ARK, mutation to the basic amino acids Arg or Lys significantly reduced the phosphorylation level by ARK (Supplementary Fig. 7c). Thus the evolutionarily conserved motif in the activation loop of subclass III SnRK2s is important for the ABA-dependent activation of the kinase through ARK. Since we observed partly different effects of the mutations between the in vitro phosphorylation assay and the in vivo functional assay, it is possible that unknown factor(s) such as a protein phosphatase is involved in the regulation of phosphorylation status of the motif.

### Evolutionarily conserved function of subclass III SnRK2

**Fig. 5:**
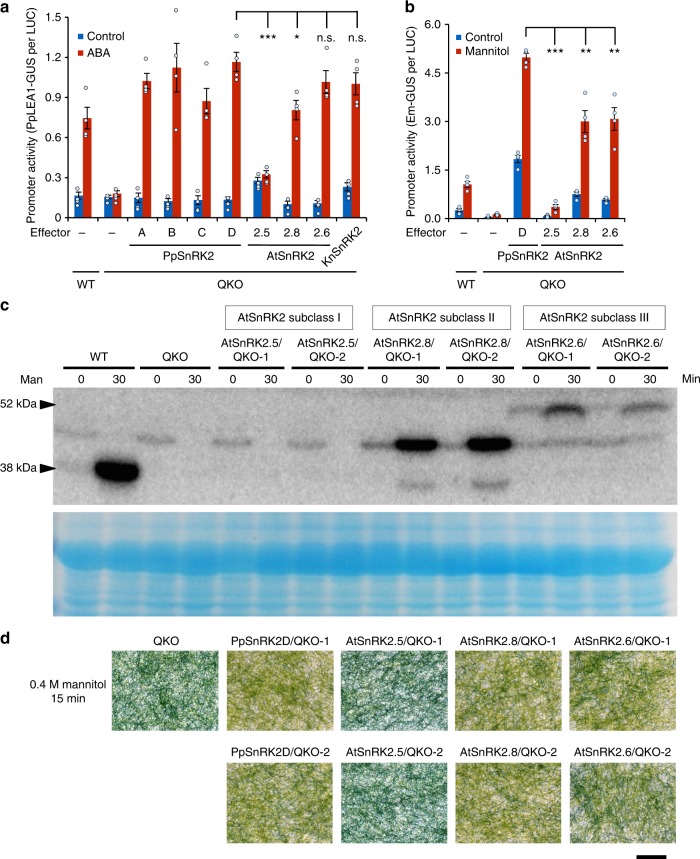
Evolutionarily conserved function of subclass III SnRK2 in ABA- and osmostress signaling. **a** *SnRK2* genes from *K. nitens*, *P. patens* and Arabidopsis were fused to the *PpSnRK2D* gene promoter and introduced into QKO protonemata with *PpLEA1-GUS* by particle bombardment. After bombardment protonemata were treated with or without 10 µM ABA for 1d. **b** *SnRK2* genes from Arabidopsis were fused to the *PpSnRK2D* gene promoter and introduced into QKO protonemata with *Em-GUS* and *Ubi-LUC* by particle bombardment. After bombardment protonemata were treated with or without 0.4 M mannitol for 1d. Levels of gene expression are represented by GUS per LUC ratio. Error bars indicate the SE (*n* = 4). *P < 0.05, **P < 0.01, ***P < 0.001 compared with the *PpSnRK2D* effector of QKO with ABA (**a**) or with mannitol (**b**). n.s., not significant. **c**, **d** Two independent transgenic *P. patens* plants that stably express Arabidopsis subclass I SnRK2.5, Subclass II SnRK2.8, or subclass III SnRK2.6 under *PpSnRK2D* gene promoter in QKO background were treated with 0.4 M mannitol for 30 min and subjected to the in-gel phosphorylation assay (**c**), or treated by 0.4 M mannitol solution for 15 min followed by Evans Blue staining to visualize the dead cells (**d**). Scale bars, 1 cm. WT, wild-type

To investigate the evolutionarily conserved function of SnRK2 in land plants, we performed a complementation assay of *SnRK2* genes within moss and cross-species complementation using a transient assay of QKO protonemata (Fig. 5a). The failure of ABA-dependent activation of *PpLEA1-GUS* in the QKO plants was recovered by co-bombardment of each *PpSnRK2* gene driven by the *PpSnRK2D* promoter. To evaluate whether SnRK2s from angiosperms could complement the function of *Physcomitrella* SnRK2, we used representative *SnRK2* genes of subclasses I (*SnRK2.5*), II (*SnRK2.8*), and III (*SnRK2.6*) from Arabidopsis as effectors in the transient assay under the control of the *PpSnRK2D* gene promoter. As a result, Arabidopsis *SnRK2* genes of subclasses II and III but not subclass I restored ABA responsiveness to the QKO plants, as judged by the *PpLEA1* promoter activity (Fig. 5a). We further tested if SnRK2 of non-land plants could rescue the function of PpSnRK2. Strikingly, the *SnRK2* of the semiterrestrial alga *K. nitens*^24^ also rescued the ABA-induced expression of *PpLEA1-GUS* in QKO (Fig. 5a). We further performed the transient assay using the *ark* null-mutant background (Supplementary Fig. 8a). Arabidopsis SnRK2.6 and 2.8 could not induce *PpLEA1-GUS* expression by ABA in the *ark* background, indicating that Arabidopsis subclass III SnRK2s also require ARK for activation by ABA in the moss. Our data suggest that ancestral *SnRK2* genes capable of functioning in ABA signalling emerged prior to the evolution of land plants.

In addition, we investigated the kinase function in response to osmostress (Fig. 5b). Arabidopsis subclasses II and III induced *Em-GUS* expression in response to osmostress, whereas subclass I SnRK2.5 again failed to activate it. The osmostress activation of Arabidopsis SnRK2s was confirmed in transgenic moss strains expressing Arabidopsis SnRK2s in the QKO background (Fig. 5c and Supplementary Fig. 8b). Arabidopsis SnRK2.8 and 2.6 but not Subclass I SnRK2s (2.4, 2.5, and 2.10) were activated by osmostress, although SnRK2 proteins were expressed similarly in the transgenic plants (Supplementary Fig. 8c). Moreover, Arabidopsis SnRK2.8 and 2.6 transformants restored the ABA sensitivity and osmotolerance of QKO mutant plants, whereas Subclass I SnRK2s did not (Fig. 5d and Supplementary Figs. 9a and 9b). Thus, these data suggested that the system for osmostress activation of SnRK2 subclasses II and III is evolutionarily conserved between the bryophyte and Arabidopsis, whereas the bryophyte does not possess the necessary upstream and/or downstream factors for subclass I SnRK2s to function in osmostress responses. Our result also agreed well with the previous report that the activation mechanism of Arabidopsis subclass III SnRK2 by osmostress is different from that of subclass I^42^.

## Discussion

Our results show the fundamental role of SnRK2 in ABA signalling as well as in osmostress and dehydration tolerance (including desiccation) of *P. patens*, an extant member of a basal land plant lineage. Furthermore, we demonstrated that even an algal SnRK2 from the evolutionarily distant charophyte, *K. nitens*, is functionally equivalent to that of land plants in executing ABA signalling, clearly identifying that the origins of SnRK2-mediated responses predate the evolution of land plants. Previous reports suggest that subclass III SnRK2 is the prototype of the SnRK2 family and that SnRK2 subclasses I and II evolved after separation of bryophytes and vascular plants, with the former representing the most recent form^6,43^. Taking advantage of the increased genome information from evolutionarily distant plant species such as charophycean algae and gymnosperms, we provided further insights into *SnRK2* gene family evolution by a survey of *SnRK2* genes from published genome sequences and expressed sequence tags from algae and land plants (Supplementary Figs. 10–14). All the charophycean algae investigated so far (but none of the chlorophytes) possess only a single *SnRK2* gene that belongs to subclass III (Supplementary Fig. 10). In bryophytes, the mosses *Sphagnum fallax* and *Physcomitrella patens*, and the liverwort *Marchantia polymorpha*, also have only subclass III *SnRK2* genes (Supplementary Fig. 11). The other subclasses of *SnRK2* are detected only in vascular plants (Supplementary Figs. 12 and 13). Thus it is likely that the precursor of the *SnRK2* genes (subclass III-type) arose before the evolution of land plants, and that gene duplication and subfunctionalisation (the emergence of subclasses I and II) occurred after the separation of bryophytes and vascular plants (Supplementary Fig. 14). The land plants are monophyletic in origin, indicating descent from a single successful colonisation of the terrestrial environment by an aquatic ancestor within the charophyte algae approximately 500 million years ago^44^. Phylogenomic analyses have supported the identification of either the Charales^45^ or the Zygnematales^46^ as sister to the land plants, with the argument provided by Wickett et al.^46^ favouring the Zygnematales currently the most convincing, based as it is on phylogenetic reconstruction from the extensive sequence data generated in the 1000 plants programme. A number of extant charophyte species occupy semiterrestrial habitats, supporting the hypothesis that the successful colonisation of land derived from algal adaptations that enabled these anatomically simple algae to survive the challenging environmental conditions encountered on land^47–49^. Bryophytes are thought to be the most ancient extant land plant lineages, and the ability to tolerate dehydration must have been a key trait enabling successful colonization of land, and in land plants this trait is regulated by ABA. Significantly, although charophyte algae do not respond to ABA^50^, the Klebsormidium genome shares with the land plants all the core genes of ABA signalling with the exception of ABA receptors^24^, and our observation that the *Klebsormidium* SnRK2 ortholog can complement the *P. patens* QKO mutant in transducing an ABA signal supports the view that the recruitment of ABA to regulate a pre-existing ARK-SnRK2-dependent dehydration response was a major adaptation enabling the successful colonisation of terrestrial environments. It is evident from the transient assay that orthologs of ABA receptors (PYR/PYL/RCAR) in *P. patens* function in ABA signalling upstream of SnRK2 since their introduction into wild-type cells enhanced the ABA response of the *PpLEA1* promoter but no effect was observed when introduced into QKO (Supplementary Fig. 15). Although the activation of Arabidopsis SnRK2 is regulated through ABA-dependent interaction between ABA receptors and PP2CA, *P. patens* PP2CA seems to act downstream of SnRK2^20^. Recent phosphoproteomic analysis of *P. patens* demonstrated that SnRK2 was phosphorylated in an ABA-dependent manner in the absence of PP2CA^35^, indicating the existence of an additional ABA-signal transduction pathway independent of the PYL/PP2CA interaction. These observations suggest an ancestral form of ABA signalling, in which ABA-dependent activation of SnRK2 is mediated through ARK. ARK belongs to the B3 mitogen-activated protein kinase kinase kinase (MAPKKK) family, which can be divided into three subgroups (ARK-like, CONSTITUTIVE TRIPLE RESPONSE1-like, and ENHANCED DISEASE RESISTANCE-like), but ARK-like B3- MAPKKK was lost during land plant evolution^21^, probably by acquiring a new mechanism to regulate subclass III SnRK2 by PP2CA through the PYL-ABA interaction. Emergence of a novel subclass I SnRK2 signalling module after the separation of bryophytes and vascular plants might have enabled vascular plants to develop new systems to cope with osmostress conditions independent of ABA/PP2CA regulation.

## Methods

### Plant materials and growth condition

The Gransden wild-type strain of *Physcomitrella patens* ssp. patens was used as the wild-type strain. Protonemal tissue was grown on cellophane-layered BCDAT medium (1.84 mM KH_2_PO_4_ [pH 6.5], 10 mM KNO_3_, 1 mM MgSO_4_, 1 mM CaCl_2_, 45 µM FeSO_4_, 10 µM H_3_BO_3_, 2 µM MnCl_2_, 0.22 µM CuSO_4_, 0.23 µM CoCl_2_, 0.19 µM ZnSO_4_, 0.1 µM Na_2_MoO_4_, 0.17 µM KI, 5 mM ammonium tartrate, 0.8 % agar) at 25 °C under continuous light (50–80 µmol m^−2^ s^−1^). *P. patens* protonemata grown on a cellophane sheet were randomly subjected to experiments.

### Gene targeting of *PpSnRK2* genes

To create the targeting constructs for *PpSnRK2B (Pp3c6_16600)*, *PpSnRK2C (Pp3c6_11090)*, and *PpSnRK2D (Pp3c5_17150)*, regions of about 2 Kbp upstream and downstream of the open-reading frames were amplified by PCR using KOD Plus Neo (TOYOBO). The amplified fragments were verified by sequencing and cloned into p35S-loxP-BSD *(PpSnRK2B)*, p35S-loxP-HPT *(PpSnRK2C)*, or p35S-loxP-Zeo *(PpSnRK2D)*. p35S-loxP-BSD and p35S-loxP-Zeo were a generous gift from Mitsuyasu Hasebe, National Institute for Basic Biology, Okazaki, Japan. The resulting plasmids were used for generating *PpSnRK2* disruption mutants. The *PpOST1/SnRK2A (Pp3c5_21160)* disruptant (SKO)^14^ was used to establish a *ppsnrk2a/2b* double disruptant (DKO) by transforming the *PpSnRK2B* targeting construct by polyethylene glycol (PEG)-mediated protoplast transformation^51^ with 30 µg mL^−1^ blasticidin S for the screening. The resulting DKO plant was used for generating the *Ppsnrk2a/2b/2c* disruptant (TKO) with 25 µg mL^−1^ hygromycin for the screening, then the TKO plant was used for generating *Ppsnrk2a/2b/2c/2d* (QKO) with 50 µg mL^−1^ Zeocin. Disruption of *SnRK2* genes was confirmed by genomic PCR using the specific primer sets (Supplementary Fig. 1b). The DKO plant was transformed with the *PpSnRK2D* targeting construct for generating the *Ppsnrk2a/2b/2d* (ABD-TKO).

For complementation of *SnRK2* genes, the hygromycin-resistance cassette was removed from QKO plants. Protoplasts of QKO plants were transiently transformed by a *Cre*-expressing cassette (generous gift from Mitsuyasu Hasebe, National Institute for Basic Biology, Okazaki, Japan) by PEG-mediated transformation as described above. The protoplasts were grown on regenerating media without selection. A portion of regenerated filaments was transferred onto hygromycin-containing media to confirm the deletion of the hygromycin-resistance cassette. This QKO (-hyg) was used for PEG-mediated transformation by the constructs to obtain the complemented lines.

### In-gel phosphorylation assay

One-week-old protonemata of wild-type or *PpSnRK2* disruptants were treated with 10 µM ABA or 0.4 M mannitol for indicated periods and subjected to in-gel phosphorylation assays. Tissues were powdered using a Tissuelyser (QIAGEN) in liquid nitrogen and homogenized in extraction buffer containing 50 mM β-glycerophosphate, 50 mM HEPES pH 7.5, 5 mM EDTA, 2 mM PMSF, 5 mM EGTA, 2 mM DTT, 1 mM Na_3_VO_4_, 25 mM NaF, 2 mg ml^−1^ leupeptin, 2 mg ml^−1^ pepstatin A, and 20% glycerol. Equal amounts of total soluble proteins were electrophoresed in 10% SDS-polyacrylamide gel containing 0.5% (w v^−1^) histone IIIS as substrates. After denaturation and renaturation procedures, the kinase activity was detected by incubation with 50 µM γ-^32^P-labeled ATP in a buffer containing 2 mM DTT, 40 mM Hepes-KOH (pH 7.5), 0.1 mM EGTA and 5 mM MgCl_2_, followed by washing^20^ and visualization of the blots was done by autoradiography or BAStation 2500 (Fuji Film).

### Tests of stress tolerance

The osmostress treatment was carried out by soaking the one-week-old protonemata in indicated concentrations of mannitol solution for 15 min, then washed with water. The survival of protonemal cells was checked by Evans Blue staining or cultured on BCDAT medium without mannitol for one week. For the evaluation of dehydration tolerance, protonemata were treated with or without ABA for 24 h, then transferred to an empty Petri dish and dehydrated inside a sealed chamber in atmospheres of known relative humidity (RH), generated by saturated salt solutions; these were placed in an incubator at 25 °C with a continuous light for 24 h. These tissues were then rehydrated by incubating under the standard growth condition described above for two weeks. Saturated salt solutions were used to generate constant RH at 25 °C and the corresponding water potential values were 75.3% RH and −39 MPa for NaCl and 95% RH and −4 MPa for K_2_SO_4_, respectively^17^.

### Protein analysis

Protonemata were incubated either with or without 1 and 10 µM ABA, or 0.25 and 0.5 M mannitol for one day. Boiling-soluble fractions of the extracted proteins were separated by SDS-polyacrylamide gel and stained with Coomassie Brilliant Blue. Boiling-soluble protein fractions were subjected to immunoblot analysis using antibody against the LEA-like protein 17B9. The antibody-reacted proteins were visualized by reaction with the chemiluminescent reagent and exposure to X-ray film. The anti-17B9 (anti-rabbit antiserum against recombinant 17B9 protein purified from *E. coli*) and the anti-rabbit IgG HRP (#458, MBL Life science, Japan) were used at 1:1000 and 1:10000 dilutions, respectively. All full-length blots and gels are shown in Supplementary Figures 16–23.

### Transient assay

Each of the coding sequences for *PpSnRK2* genes, for *KnSnRK2* or for Arabidopsis *SnRK2.5*, *SnRK2.6*, and *SnRK2.8* were cloned into the expression cassette controlled by the *PpSnRK2D* native promoter^51^. These plasmids were subjected to the transient assay using particle bombardment of protonemata^51^. We used 0.8 µg each of the reporter constructs (*Em-GUS* or *PpLEA1-GUS* and *Ubi-LUC*) and the effector construct to prepare DNA-coated gold particles for four shots. One-week-old protonemal tissue was used and incubated on BCDAT agar medium with or without 10 µM ABA or 0.4 M mannitol for 24 h. GUS activity was normalized by the luciferase activity and represented as relative GUS activity (GUS per LUC).

### Transcriptomic analysis

Protonemata were cultured for one week under normal conditions as described above. For osmostress and ABA treatments, the protonemata were transferred to fresh medium, supplemented with 0.4 M mannitol, while for ABA treatments, ABA was added to a final concentration of 10 µM. After 12 h, the protonemata were harvested. Total RNA was extracted from the protonemal tissues using the RNeasy plant mini Kit (QIAGEN). The RNA-seq libraries were prepared using the TruSeq RNA sample prep kit v2 (Illumina) in accordance with manufacturer’s instructions. Library quality control was performed with a Bioanalyzer 2100 (Agilent). The libraries included inserts that ranged in size from ~200–500 bp. The average insert size in the library was approximately 300 bp. Library quantification was performed with quantitative real-time PCR, and then the concentration of each library was adjusted to 10 nM. The prepared libraries were sequenced using the Illumina HiSeq 2500 system (Illumina). The raw reads for each library were deposited in DDBJ and are accessible through Sequence Read Archive accession number DRA005879. Paired-end reads from each library were processed using CASAVA version 1.8.2 in FASTQ format. The FASTQ files were imported to the CLC Genomics Workbench (QIAGEN) for the following analysis. The trim sequences tool in the suite was used to remove adapters and filter out low-quality bases ( < Q13), and only those reads that showed a quality score of 13 or higher were retained. Filtered sequence reads were mapped onto the *P. patens* genome v3.3 (Phytozome 11; https://phytozome.jgi.doe.gov) using CLC assembly with default parameters. Quality control was carried out with filtering for the number of mapped reads in the exon region; genes with fewer than 5 mapped reads in any samples were removed. After filtering, a total of 16,924 genes were subjected to further analysis. Expression values were reported as RPKM values. Differentially expressed genes were detected by empirical analysis with the DGE algorithm in CLC software. The results were filtered based on a false discovery rate (FDR) with P values less than 0.05 and a corrected fold change greater than 2. Principal component analysis (PCA) was performed using the “Principal component analysis” function in CLC software, and the results are shown as a scatterplot with principal components as the axes. Hierarchical clustering and heatmap analysis were performed based on Euclidian distance in the CLC software. The RPKM values were used for both PCA and the hierarchical clustering analysis. Gene ontology enrichment analysis was performed using BiNGO (Biological Network Gene Ontology) software, a Cytoscape plug-in (available at www.cytoscape.org) for putative Arabidopsis orthologues of SAGs. These putative Arabidopsis orthologues were identified by a blastp search against the TAIR10 protein database. The degree of functional enrichment for a given cluster was quantitatively assessed by a hypergeometric test, and a multiple test correction was applied using the FDR algorithm implemented in the BiNGO plug-in. Overrepresented gene ontology terms were generated after FDR correction, with a significance level of 0.05.

### Phosphoproteomic analyses

*P. patens* wild type and *SnRK2* disrupted mutants were treated with 10 μM ABA for 0, 15, 30 and 90 min. Plant samples were homogenized in protein extraction buffer containing 10 mM Tris-HCl (pH 9.0), 8 M Urea, 2% Phosphatase Inhibitor Cocktail II (Sigma) and 2% Phosphatase Inhibitor Cocktail III (Sigma), and then centrifuged at 15,000 r.p.m for 10 min on 4 °C to obtain crude extracts. Aliquots of crude extract (500 μg of total protein) were subjected to tryptic digestion and phosphopeptide enrichment with HAMMOC^34^. Enriched phosphopeptide samples were analyzed with a LC-MS/MS system, TripleTOF 5600 (SCIEX). Peptides and proteins were identified using the *Physcomitrella patens* database (Phytozome v12.1) with Mascot (Matrix Science)^34^. Skyline v3.7 (Maccoss lab software) was used for quantification of phosphopeptides on the basis of LC-MS peak area. The motif analysis was conducted using the Motif-X algorithm^52^.

### Primers

Primer sequences used in this study are described in Supplementary Data 8.

### Statistical analysis

Four biological repeats except for Fig. 4b (three biological replicates) were conducted for the transient assays. Statistical significance between two samples was analyzed by two-tailed Student’s *t* test. The experiments were repeated at least three times on different occasions to confirm the reproducibility, and the representative experiments were presented. Error bars indicate the standard error. *P < 0.05, **P < 0.01, ***P < 0.001.

### Southern blot analysis

The genomic DNA of the *Ppsnrk2* plants was extracted using PhytoPure (GE Healthcare) with an additional RNase treatment. The probes were amplified using a PCR DIG Probe Synthesis Kit (Roche Applied Science) with primers described in Suppelemtary Data 8. Two µg of genomic DNAs were digested with restriction enzymes and were separated on 0.6% agarose gels and transferred to nylon filters (Hybond-N, GE Healthcare). The membrane was hybridized with DIG-labelled probes in DIG Easy Hyb buffer (Roche Applied Science) at 41 °C overnight. After washing, the membrane was treated once with blocking buffer for 1 h at room temperature. The membrane was reacted with Anti-Dig (Roche Applied Science) for 1 h at room temperature. Detection was carried out with CDP-Star (Roche Applied Science) for 5 min at room temperature in the dark. The signal was detected by ChemiDoc XRS Plus (BIO-RAD).

### Quantitative RT-PCR

First-strand complementary DNA was synthesized from 0.5 ug total RNA subjected to RNA-seq analysis using a ReverTra Ace qPCR RT Master Mix with gDNA Remover (TOYOBO). qRT-PCR was performed using LightCycler 96 (Roche Diagnostics). THUNDERBIRD SYBR qPCR Mix (TOYOBO) was used for amplification. Ubiquitin-conjugating enzyme E2 (Pp3c14_21480) was used as an internal standard. Primers are listed in Supplementary Data 8.

### In vitro phosphorylation assay

For in vitro phosphorylation assay of PpSnRK2D by ARK, cDNA of *PpSnRK2D* with or without mutations was subcloned into the pMAL-c5X vector (New England Biolabs) and expressed in *Escherichia coli* (BL21). The MBP-fused PpSnRK2 proteins were purified by amylose resin. The vector expressing ARK-KD^23^ was expressed in *Escherichia coli* (BL21). GST-fused ARK-KD protein was purified by glutathione-Sepharose resin. Both recombinant proteins were subjected to ultrafiltration using a Nanosep 30-kDa size-exclusion column (Pall) for concentration and removal of low-molecular-weight materials. In vitro phosphorylation reactions were carried out using the recombinant proteins by incubating at 30 °C for 30 min in the reaction buffer^23^ in the presence of γ−32P ATP. The reaction mixture was separated by SDS-PAGE and phosphorylated protein detected by autoradiography. For in vitro phosphorylation assay of ABA-and PpSnRK2-regulated phosphopeptides by PpSnRK2B, we selected 3 peptides, of which phosphorylation levels were decreased in ABA-treated QKO in phosphoproteome analysis and designed primers to synthesise 33 amino acid sequences in total for each peptide with a phosphorylation site near the center, and GST-fused proteins were prepared using pGEX-5X-3 vector in *Escherichia coli* (KRX). The GST-fusion peptides were purified as described above, and GST protein was used as a negative control.

### Immunoblot assay

Total proteins from *P. patens* wild-type and *Ppsnrk2* plants were extracted with buffer (50 mM HEPES pH 7.5, 5 mM EDTA, 5 mM EGTA, 25 mM NaF, 2 mM DTT, 1 mM Na_3_VO_4_, 2 mg ml^−1^ leupeptin, 2 mg ml^−1^ pepstatin A, 2 mM PMSF, 50 mM b-glycerophosphate, and 20% glycerol). Equal amounts of total soluble proteins were electrophoresed in 10% SDS-polyacrylamide gel. The proteins were detected by the anti-HA-tag pAb (#561, MBL Life science). Detection of the antibody-reacted proteins were carried out with Amersham ECL Prime Western Blotting Detection Reagent (GE Healthcare). The signal was detected by ChemiDoc XRS Plus (BIO-RAD). The anti-HA-tag pAb and the anti-rabbit IgG (H + L) (#170–6515, BIO-RAD) were used at 1:2000 and 1:50000 dilutions, respectively.

### RNA blot analysis

Total RNAs were extracted from protonemal tissues of wild-type and *ppsnrk2* plants treated with or without 10 µM ABA for indicated periods using the RNeasy plant mini Kit (QIAGEN). The primer sets of probes are described in Supplementary Data 8. Hybridization was carried out with ^32^P-labelled DNA probes. The signal was detected by BAStation 2500 (Fuji Film).

### Measurement of freezing tolerance

One-week old protonemata of wild-type and *Ppsnrk2* plants were treated with or without 10 µM ABA for 24 h and subjected to freezing at −5 °C. Freezing tolerance was determined by measurement of electrolyte leakage. The amount of electrolyte leakage from the freeze-thawed tissues (Eft) and the amount of electrolytes after boiling (Eb) were measured. Survivals of the cells were represented as the Eft per Eb ratios (%).

## Supplementary information

Description of Additional Supplementary Files

Supplementary Information

Supplementary Data 1

Supplementary Data 2

Supplementary Data 3

Supplementary Data 4

Supplementary Data 5

Supplementary Data 6

Supplementary Data 7

Supplementary Data 8

Supplementary Data 9

Supplementary Data 10

## Data Availability

*Physcomitrella* Sequence data of *SnRK2* genes and other genes used in this study can be found from Phytozome (DOE-JGI, http://phytozome.jgi.doe.gov/). The RNA-seq data are deposited to DDBJ Sequence Read Archive (DRA) with the accession number DRA005879. All raw data files for phosphoproteome were deposited in the Japan Proteome Standard Repository/Database (jPOST; PST000493). Data source of amino acid sequences of SnRK2-type protein kinases used for phylogenetic analysis is available in Supplementary Data 9. Source data for all figures presented in the manuscript are available in Supplementary Data 10. All other data supporting the results of this study are available from the authors upon request.
