# Publisher Correction: SnRK2 protein kinases represent an ancient system in plants for adaptation to a terrestrial environment

**DOI:** 10.1038/s42003-019-0312-y

**Published:** 2019-02-04

**Authors:** Akihisa Shinozawa, Ryoko Otake, Daisuke Takezawa, Taishi Umezawa, Kenji Komatsu, Keisuke Tanaka, Anna Amagai, Shinnosuke Ishikawa, Yurie Hara, Yasuko Kamisugi, Andrew C. Cuming, Koichi Hori, Hiroyuki Ohta, Fuminori Takahashi, Kazuo Shinozaki, Takahisa Hayashi, Teruaki Taji, Yoichi Sakata

**Affiliations:** 1 Department of Bioscience, Tokyo University of Agriculture, 1-1-1 Sakuragaoka, Setagayaku, Tokyo, 156-8502 Japan; 2 Graduate School of Science and Engineering, Saitama University, 255 Shimo-Okubo,Sakura-ku, Saitama, 338-8570 Japan; 3 Graduate School of Bio-Applications and Systems Engineering, Tokyo University of Agriculture and Technology, 2-24-16 Nakacho, Koganei, Tokyo, 184-8588 Japan; 4 Department of Bioproduction Technology, Junior College, Tokyo University of Agriculture, 1-1-1 Sakuragaoka, Setagayaku, Tokyo, 156-8502 Japan; 5 NODAI Genome Research Center (NGRC), Tokyo University of Agriculture, 1-1-1 Sakuragaoka, Setagayaku, Tokyo, 156-8502 Japan; 6 Centre for Plant Science, University of Leeds, Leeds, LS2 9JT UK; 7 School of Life Science and Technology, Tokyo Institute of Technology, 4259 Nagatsuta-cho, Midori-ku, Yokohama, Kanagawa 226-8503 Japan; 8 Gene Discovery Research Group, RIKEN Center for Sustainable Resource Science, 3-1-1, Koyadai, Tsukuba, Ibaraki, 305-0074 Japan

Correction to: *Communications Biology* **2**:30 10.1038/s42003-019-0281-1, published online: 21 January 2019

In the original published PDF version of the article, the labels on the graphs in Fig. 2c were inadvertently swapped. The error has been corrected in the PDF.

