# Supplementary material for: SnRK2 protein kinases represent an ancient system in plants for adaptation to a terrestrial environment
Source: Commun Biol. 2019 Jan 21;2:30. doi: 10.1038/s42003-019-0281-1 (PMC6340887; doi:10.1038/s42003-019-0281-1)
Supplement: Supplementary file 1 — Description of Additional Supplementary Files [file 42003_2019_281_MOESM1_ESM.docx]

**Description of Additional Supplementary Files**

**File Name**: Supplementary Data 1

**Description**: ABA and osmostress responsive genes and differentially expressed genes in *ppsnrk2*.

**File Name**: Supplementary Data 2

**Description**: Genes list regulated in common by SnRK2 between Arabidopsis and *P. patens*.

**File Name**: Supplementary Data 3

**Description**: Salvaged ABA, Osmostress and SnRK2 regulated genes in *P. patens.*

**File Name**: Supplementary Data 4

**Description**: List of phosphopeptides detected in this study.

**File Name**: Supplementary Data 5

**Description**: List of SnRK2-dependet or ABA-upregulated phosphopeptides of *P. patens*.

**File Name**: Supplementary Data 6

**Description**: A list of phosphopeptides for in vitro phosphorylation assay.

**File Name**: Supplementary Data 7

**Description**: Classification of selected phosphopeptides by motif groups.

**File Name**: Supplementary Data 8

**Description**: Primer sequences used in this study.

**File Name**: Supplementary Data 9

**Description**: Data source of amino acid sequences of SnRK2-type protein kinases used for phylogenetic analysis.

**File Name**: Supplementary Data 10

**Description**: Source data of figures in this study.
