# Supplementary material for: SnRK2 protein kinases represent an ancient system in plants for adaptation to a terrestrial environment
Source: Commun Biol. 2019 Jan 21;2:30. doi: 10.1038/s42003-019-0281-1 (PMC6340887; doi:10.1038/s42003-019-0281-1)
Supplement: Supplementary file 2 — Supplementary Information [file 42003_2019_281_MOESM2_ESM.pdf]

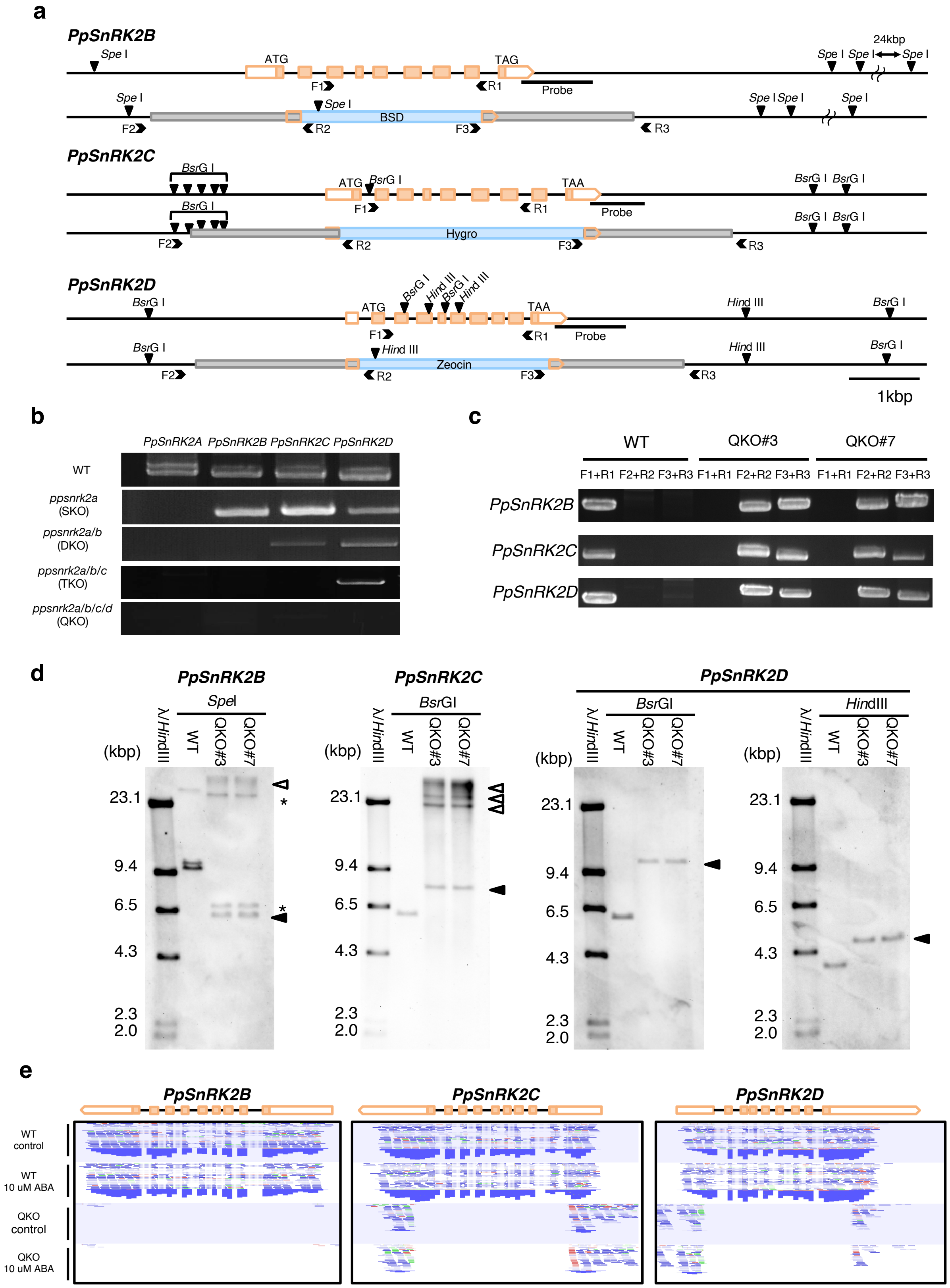

Supplementary Figure 1 (Shinozawa et al.)

Supplementary Figure 1 | Genetic characterization of *Ppsnrk2* disruptants

a, Schematic representation of *PpSnRK2* genes structures before and after homologous recombination with the targeting constructs. Primer sites used in PCR, restriction enzyme sites and probe region for Southern blot analysis are indicated. Orange-lined open boxes and shaded gray boxes indicate the exons and homologous regions used in the targeting constructs, respectively. **b**, PCR amplification of *PpSnRK2* open reading frames in SKO, DKO, TKO and QKO plants using primer sets F1+R1 shown in **a**. **c,** Confirmation of homologous recombination at the *PpSnRK2* gene loci in QKO plants. See **a** for the position of primers. **d,** Southern blot analysis of the copy numbers of the targeting constructs. Black arrowheads and white arrowheads indicate the targeted integration and off-target integration of the constructs, respectively. *; non-specific bands. In addition to the single-copy targeted gene replacement in the SKO plant[1], we confirmed by Southern blot analysis the low-copy insertion of the target constructs for *PpSnRK2B* and *PpSnRK2C*, and single-copy insertion for *PpSnRK2D* in QKO plants. Because we did not detect any significant phenotype in TKO plants, we concluded that the single-copy disruption of *PpSnRK2D* gene in TKO plants resulted in the phenotypes of QKO plants. We also performed complementation experiments to introduce *PpSnRK2* genes under the *actin* promoter into QKO plants. Resultant transgenic plants recovered ABA sensitivity (see Supplementary Fig. 2**e,f)**. Therefore, we concluded that the phenotypes we observed in QKO plants are due to the loss of *SnRK2* genes. **e,** Confirmation of null expression for *PpSnRK2* genes in the QKO plant (#7) with or without ABA treatment by RNA-seq data. No reads were detected from *PpSnRK2B, C* and *D* open reading frames in the QKO plant.

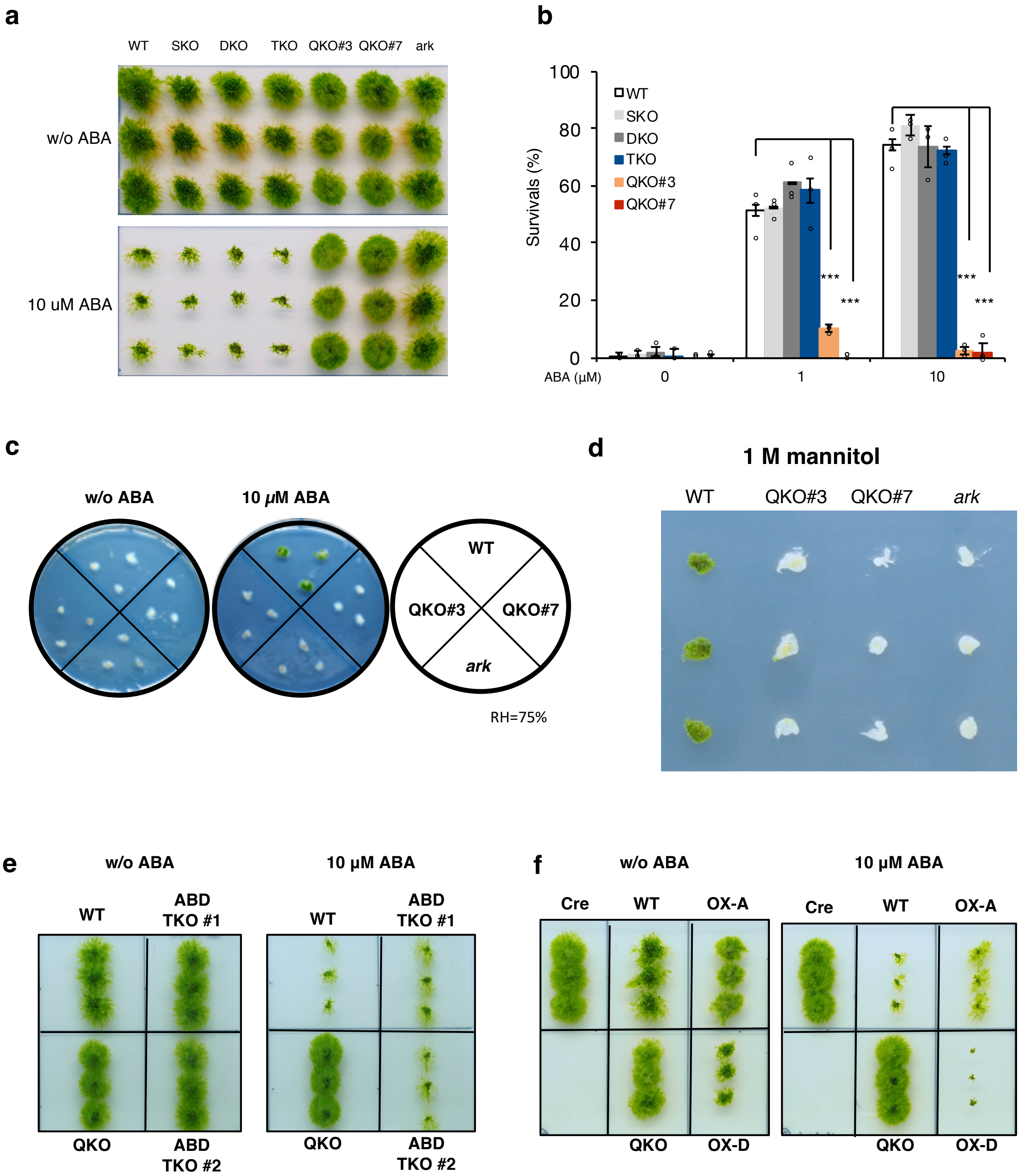

Supplementary Figure 2 (Shinozawa et al.)

Supplementary Figure 2 | ABA sensitivity and osmostress tolerance of *Ppsnrk2* disruptants

a, Protonemal growth of WT, *Ppsnrk2* SKO, DKO, TKO, and QKO, and ABA-insensitive *ark* mutant on agar media with or without ABA. Only *Ppsnrk2* QKO plants and the *ark* mutants showed ABA-insensitive growth. **b,** ABA-induced freezing tolerance of protonemata of *SnRK2* disputants. Protonemata were treated for 24 h with indicated concentrations of ABA and subjected to freezing at -5°C. Survival was evaluated by measuring the electrolyte leakage from the injured cells. Error bars indicate the standard error (SE; n = 3). ***P < 0.001. **c,** ABA-induced dehydration tolerance of *Ppsnrk2* QKO protonemata. The ABA-insensitive mutant *ark* was used as a control. Protonemata were treated with indicated concentrations of ABA for 24 h and dehydrated in an atmosphere of relative humidity of 75% for 24 h. The dehydrated tissues were rehydrated with water and grown on normal medium for two weeks. **d,** Comparison of osmostress tolerance among WT, QKO plants, and the *ark* mutant in protonemal growth. Protonemata were inoculated on medium containing 1 M mannitol and grown for two weeks, then recovered on standard growth medium without osmostress for one week. **e,** Comparison of ABA sensitivity of protonemal growth in two independent lines of *Ppsnrk2a/b/d* triple disruptants (ABD TKO) with that of WT and QKO on media with or without 10 μM ABA. **f,** Comparison of ABA sensitivity of protonemal growth in QKO, QKO with removal of selection cassettes by Cre-lox deletion (Cre), and the Cre-deleted line transformed with *PpSnRK2A* (OX-A) or *PpSnRK2D* (OX-D) overexpression construct driven by a constitutive actin promoter on media with or without 10 μM ABA.

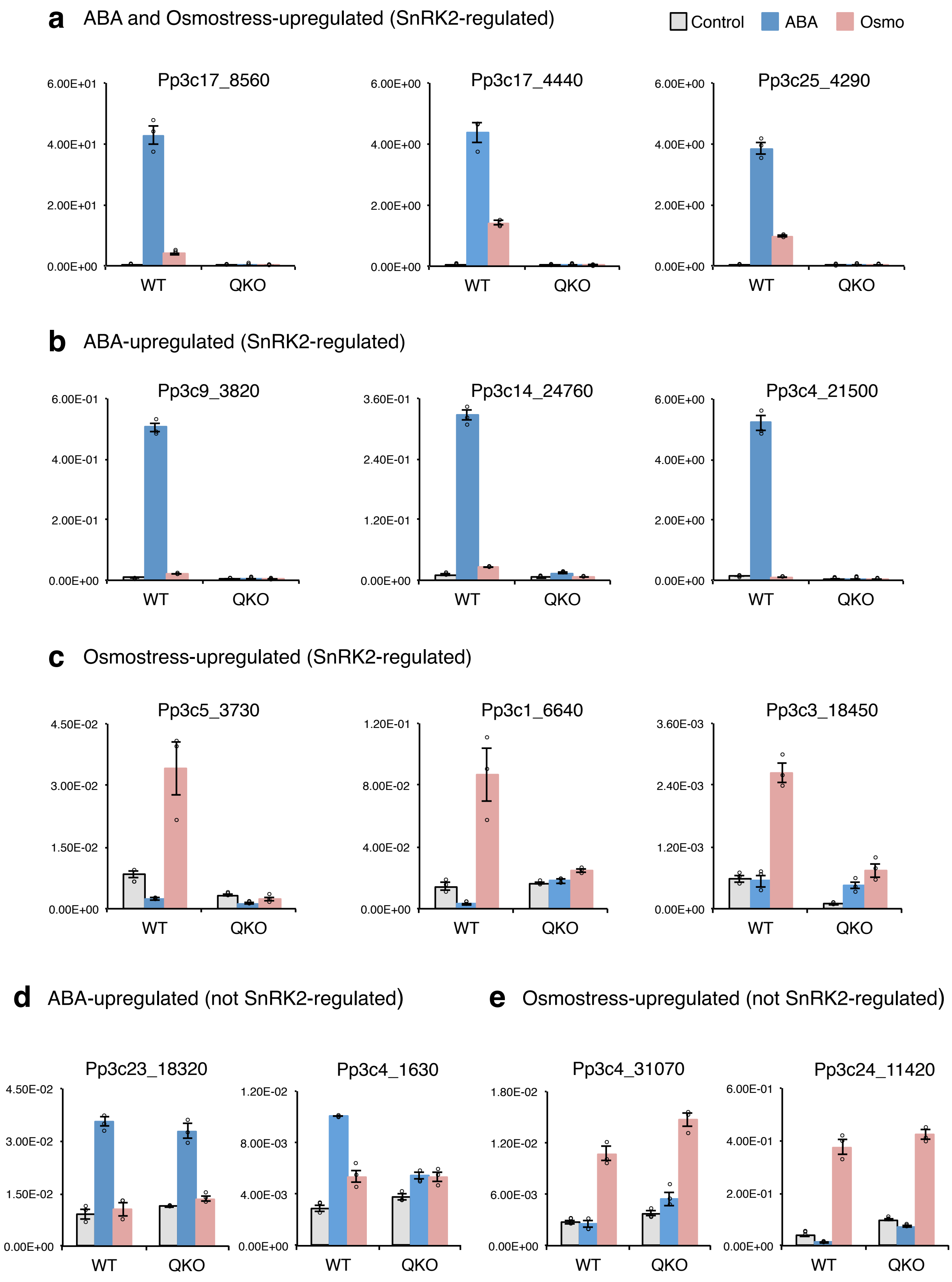

Supplementary Figure 3 (Shinozawa et al.)

Supplementary Figure 3 | Validation of RNA-seq data by qPCR

Two or three genes were selected from the categories shown in Supplementary Fig. 4a and the expression levels in response to ABA or osmostress were analyzed by qPCR using the same RNAs with the transcriptomic analysis. **a,** SnRK2-regulated ABA and osmostress-inducible genes. **b,** SnRK2-regulated ABA-inducible genes. **c,** SnRK2-regulated osmostress-inducible genes. **d,** ABA-inducible genes not regulated by SnRK2. **e,** Osmostress-inducible genes not regulated by SnRK2. The mRNA accumulatiuon of these genes was shown as a relative value to that of ubiquitin-conjugating enzyme E2 (Pp3c14_21480) used as an internal standard.

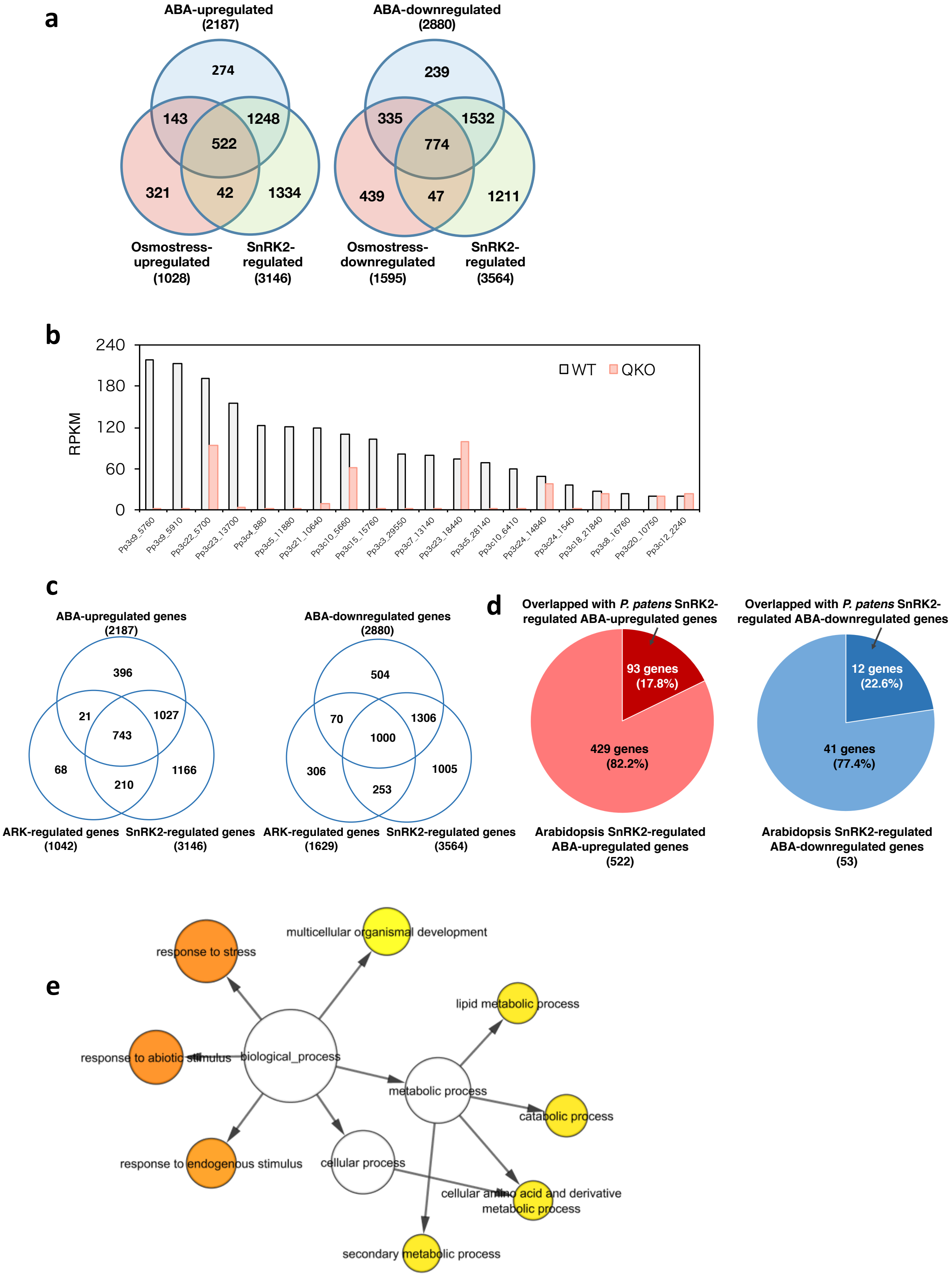

Supplementary Figure 4 (Shinozawa et al.)

Supplementary Figure 4 | Characterization of PpSnRK2-regulated genes

a, Venn diagrams show the number of upregulated genes and downregulated genes in response to ABA or osmostress in *Ppsnrk2* QKO plants compared with WT. Genes with more than two-fold difference in the expression level at any of the comparison in mock-treated WT vs QKO, ABA-treated WT vs QKO, or mannitol-treated WT vs QKO (FDR ≤ 0.05) were categorized as SnRK2-regulated. **b,** Expression level (RPKM) of 20 *LEA* genes between WT and QKO plants extracted from RNA-seq data. **c**, Venn diagrams show the number of ABA-upregulated genes and ABA-downregulated genes in response to ABA in WT, the number of ARK-regulated genes and PpSnRK2-regulated genes. **d,** Comparison of Arabidopsis SnRK2-regulated ABA-upregulated genes with *P. patens* SnRK2-regulated ABA-upregulated genes and Arabidopsis SnRK2-regulated ABA-downregulated genes with *P. patens* SnRK2-regulated ABA-downregulated genes. **e,** Gene ontology term enrichment analysis was performed against common target genes of SnRK2 in Arabidopsis and *P. patens* by BiNGO. Slim ontology was used to summarize the network.

a

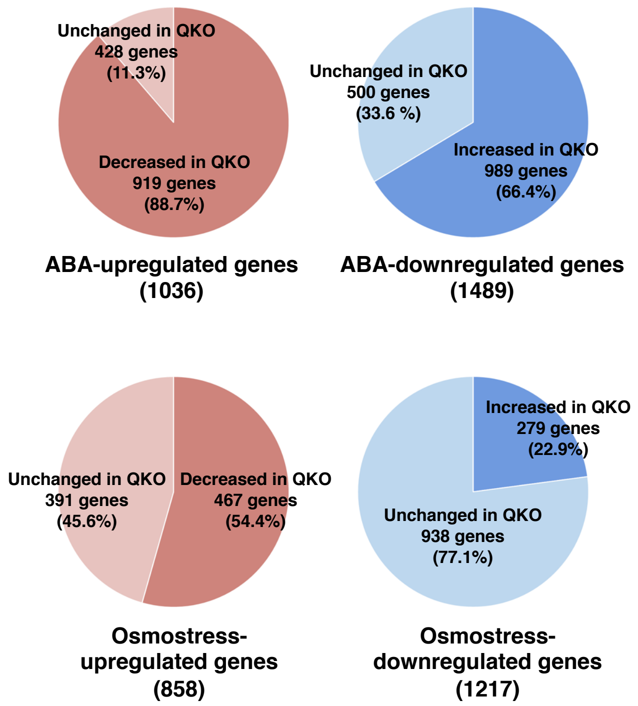

b

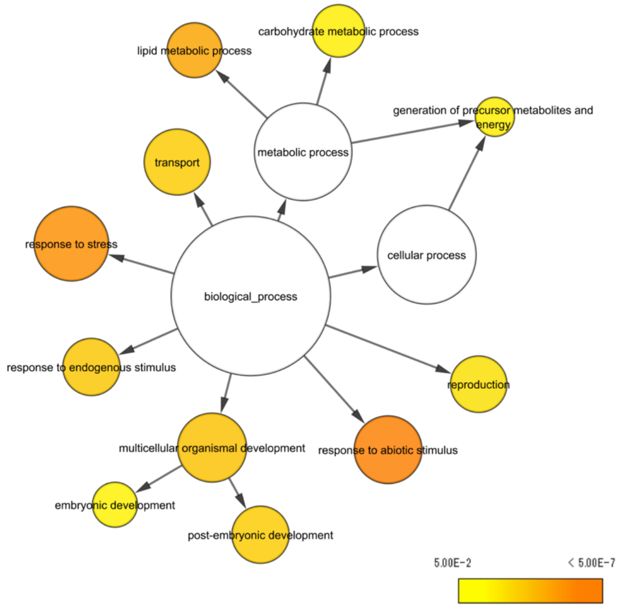

Supplementary Figure 5 (Shinozawa et al.)

Supplementary Figure 5 | Analysis of salvaged genes from WT and QKO with fewer than 5 mapped reads in any samples

Genes with fewer than 5 mapped reads in any samples of WT and QKO were analysed independently from the 16,924 genes represented by more than five mapped reads per transcript. **a,** Numbers of ABA-regulated genes and osmostress-regulated genes in WT, and the numbers of genes affected in QKO plants. **b,** Gene ontology term enrichment analysis was performed against SAGs. Functional enrichment analysis was performed for BLAST best-hit genes of SAGs, which were used to search Arabidopsis TAIR 10 proteins using the BiNGO plug-in in Cytoscape. Slim ontology was used to summarize the network.

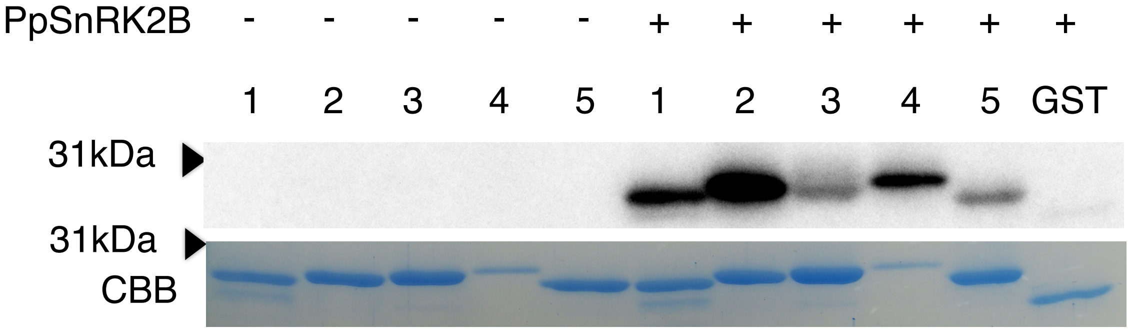

Supplementary Figure 6 (Shinozawa et al.)

Supplementary Figure 6 | *In vitro* phosphorylation assay of selected phosphopeptides.

Five phosphopeptides and PpSnRK2B were synthesized as GST-fused and MBP-fused proteins, respectively. Phosphorylation reactions were performed in reaction buffer in the presence (+) or absence (-) of PpSnRK2B. Lane number indicates each peptide as follows: No.1, Pp3c9_25200V1.1, No.2, Pp3c4_24010V3.1, No.3, Pp3c2_10380V3.1, No.4, Pp3c9_10940V3.2 and No.5, Pp3c8_2690.v3.1. Following the *in vitro* reaction, the level of phosphorylation of each substrate was visualised by BAStation 2500 (Fuji Film, Japan). Coomassie Brilliant Blue staining shows the quantity of each substrate.

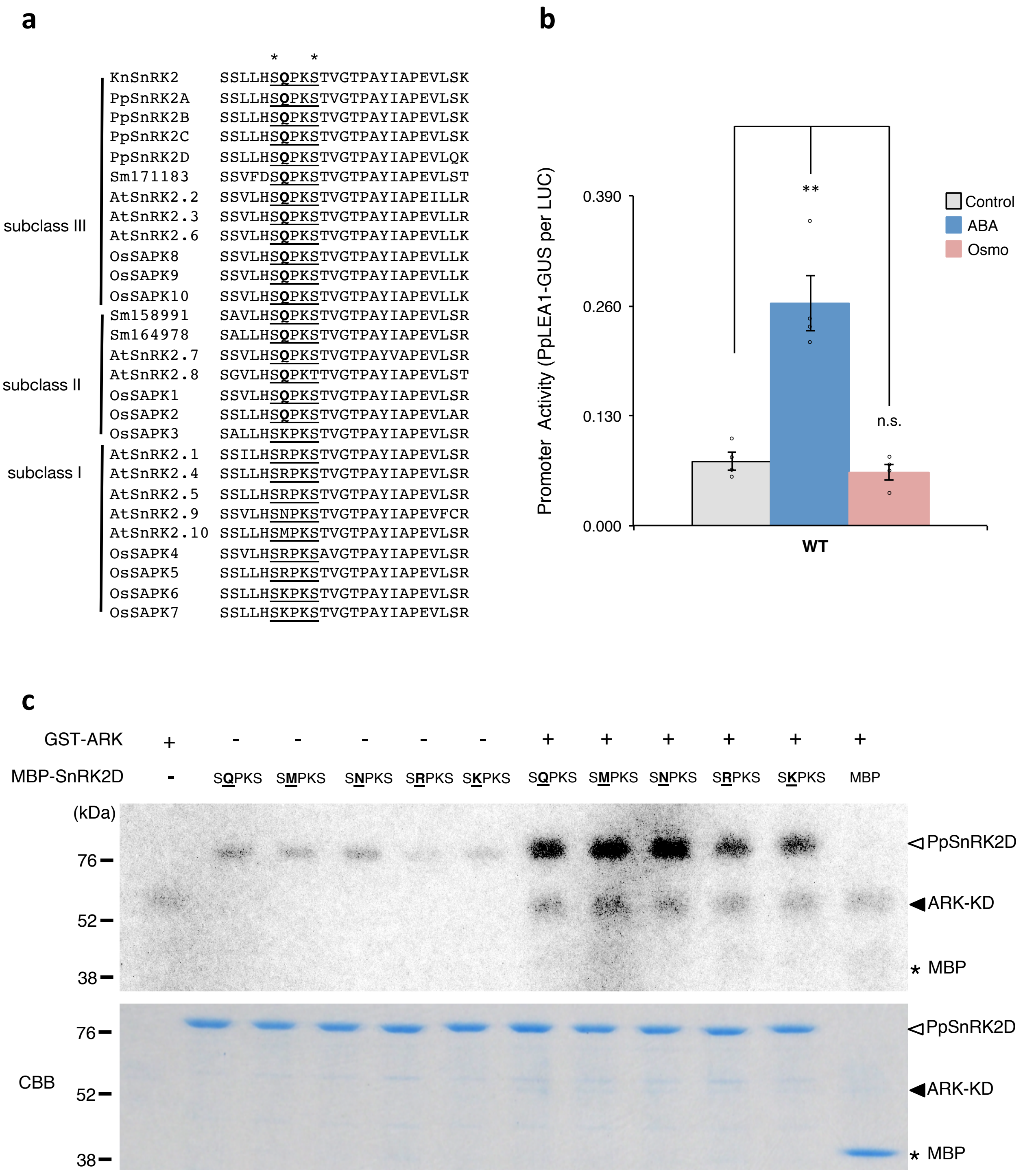

Supplementary Figure 7 (Shinozawa et al.)

Supplementary Figure 7 | Characterisation of SQPKS motif in PpSnRK2D.

a, Alignment of amino acid sequences in the activation loop of land plant SnRK2s. Comparison of amino acid sequences of the activation loop including the ARK phosphorylation target sequence (underlined) among plant SnRK2s. Kn: *K. nitens*; Pp: *P. patens*; Sm: *Selaginella moellendorffii*; At: *A. thaliana*; Os: *Oryza. sativa*. **b,** *PpLEA1-GUS* and *Ubi-LUC* constructs were introduced into WT protonemata and treated with 10 μM ABA or 0.4 M mannitol for 1d. Levels of gene expression are represented by GUS per LUC ratio. Error bars indicate the SE (n = 4). ***P*<0.01, n.s., not significant. **c,** cDNA of *PpSnRK2D* with or without mutations was subcloned into the pMAL-c5X vector (New England Biolabs) and expressed in *Escherichia coli* (BL21). The MBP-fused PpSnRK2 proteins were purified by amylose resin. The vector expressing ARK-KD[2] was expressed in *Escherichia coli* (BL21). GST-fused ARK-KD protein was purified by glutathione-Sepharose resin. Both recombinant proteins were subjected to ultrafiltration using a Nanosep 30-kDa size-exclusion column (Pall) for concentration and removal of low-molecular-weight materials. *In vitro* phosphorylation reactions were carried out using the recombinant proteins by incubating at 30°C for 30 min in the reaction buffer[2] in the presence of γ-32P ATP. The reaction mixture was separated by SDS-PAGE and phosphorylated protein detected by BAStation 2500 (Fuji Film, Japan).

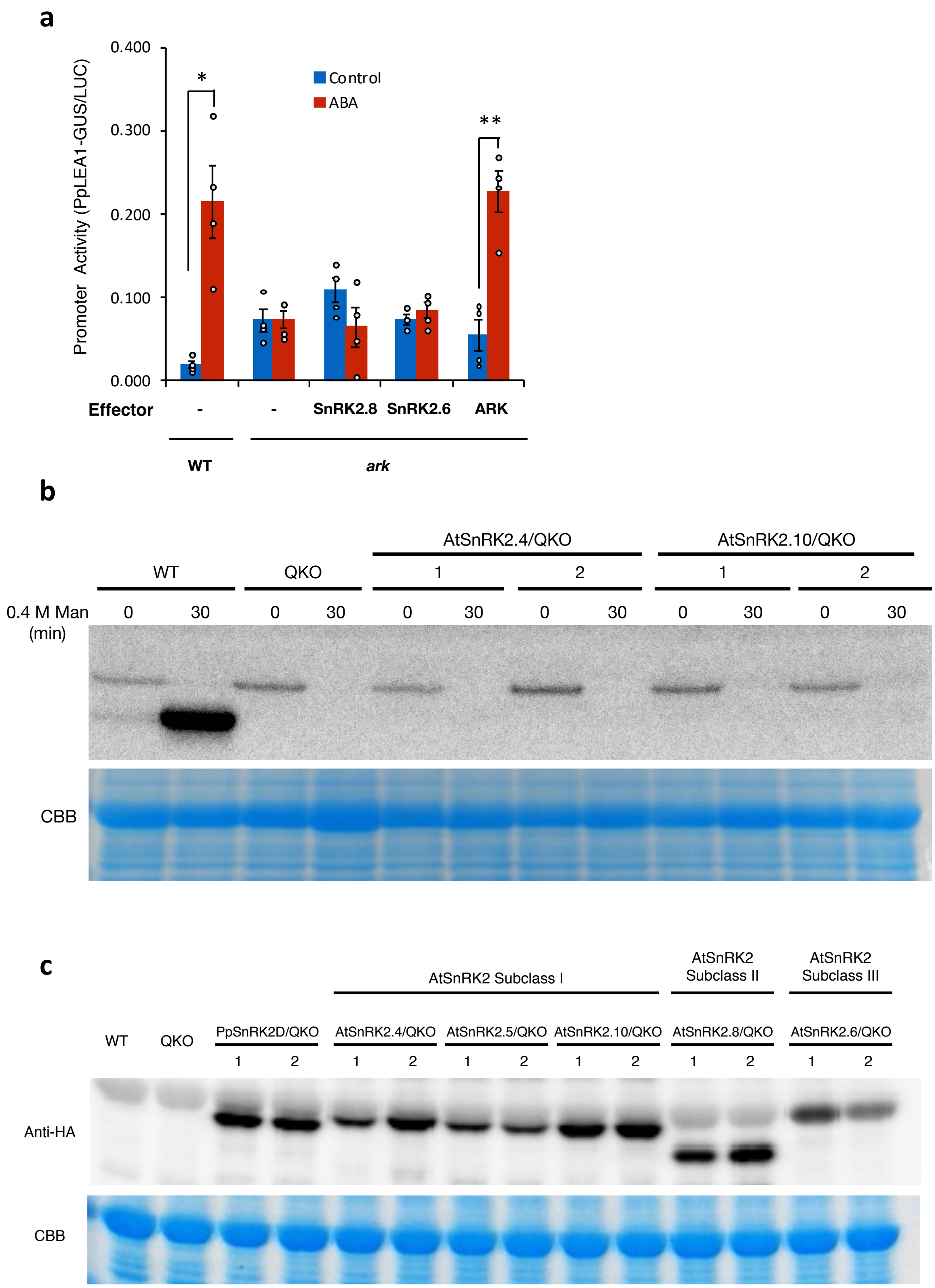

Supplementary Figure 8 (Shinozawa et al.)

Supplementary Figure 8 | Cross-species analyses of SnRK2s

a, Requirement of ARK upstream kinase for the activation of Arabidopsis SnRK2s in the moss. *SnRK2* genes from Arabidopsis were fused to the *PpSnRK2D* gene native promoter and introduced into *ark* mutant protonemata with *PpLEA1-GUS* and *Ubi-LUC* constructs and treated with 10 μM ABA for 1d. ARK was introduced as the control. Levels of gene expression are represented by GUS per LUC ratio. Error bars indicate the SE (n = 4). *P<0.05, **P<0.01. **b,** Two independent transgenic *P. patens* plants that stably express Arabidopsis subclass I SnRK2.4 or SnRK2.10 under *PpSnRK2D* gene promoter in QKO background were treated with 0.4 M mannitol for 30 min and subjected to the in-gel phosphorylation assay. CBB staining of total proteins is shown. **c,** Western blot analysis to confirm the accumulation of AtSnRK2 proteins in the two independent lines of transgenic moss plants. CBB staining of total proteins is shown.

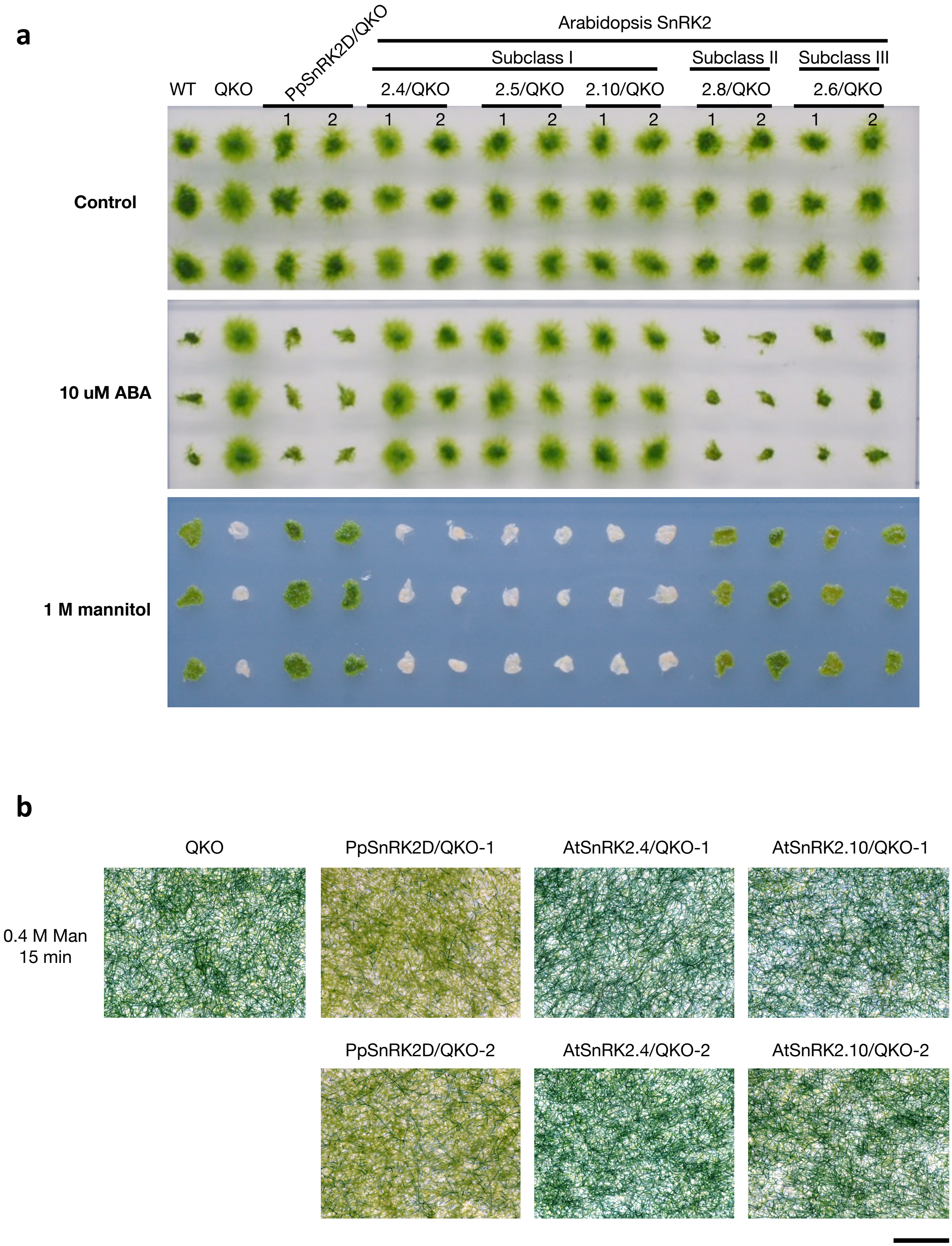

Supplementary Figure 9 (Shinozawa et al.)

Supplementary Figure 9 | Phenotypes of AtSnRK2-expressing QKO plants.

a, Two independent transgenic *P. patens* plants that stably express Arabidopsis subclass I SnRK2 (2.4, 2.5, and 2.10), Subclass II SnRK2.8, or subclass III SnRK2.6 under *PpSnRK2D* gene promoter in QKO background were grown on medium containing 10 μM ABA for 9 days. For osmostress, protonemata were grown on medium containing 1 M mannitol for two weeks followed by recovery on standard culture medium for one week. **b,** Protonemata of transgenic *P. patens* plants that stably express Arabidopsis subclass I SnRK2.4 and SnRK2.10 were incubated in 0.4 M mannitol solution for 15 min followed by Evans Blue staining to visualize the dead cells. Scale bars, 1 cm.

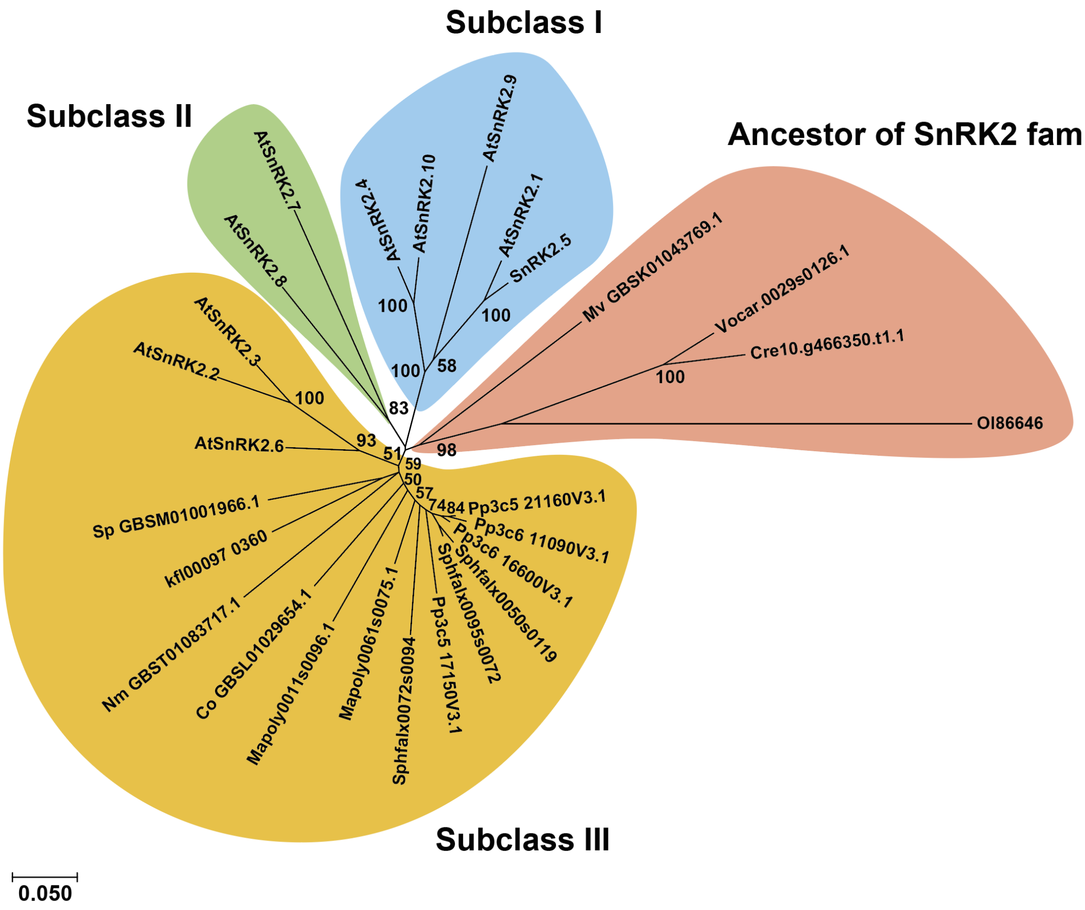

Supplementary Figure 10 (Shinozawa et al.)

Supplementary Figure 10 | Phylogram of SnRK2-type protein kinases from algaes and bryophytes.

Amino acid sequences from *Chlamydomonas reinhardtii* (Cre), *Volvox carteri* (Vocar), *Ostreococcus lucimarinus* (Ol), *Mesostigma viride* (Mv), *Klebsormidium nitens* (kf), *Nitella mirabilis* (Nm), *Colechaete orbicularis* (Co), *Spirogyra pratensis* (Sp), *Marchantia polymorpha* (Mapoly), *Sphagnum fallax* (Sphfalx), *Physcomitrella patens* (Pp), and *Arabidopsis thaliana* (At) were used for phylogenetic analysis (data source is shown in Supplementary Data 9). The sequences were aligned with ClustalW. A neighbor-joining tree was constructed based on alignment of the amino acid sequences containing the kinase and regulatory domains. The phylogram shown is unrooted. Numbers on branches indicate bootstrap values (> 50 %) from 1,000 replicates. The bar represents number of amino acid changes per branch length.

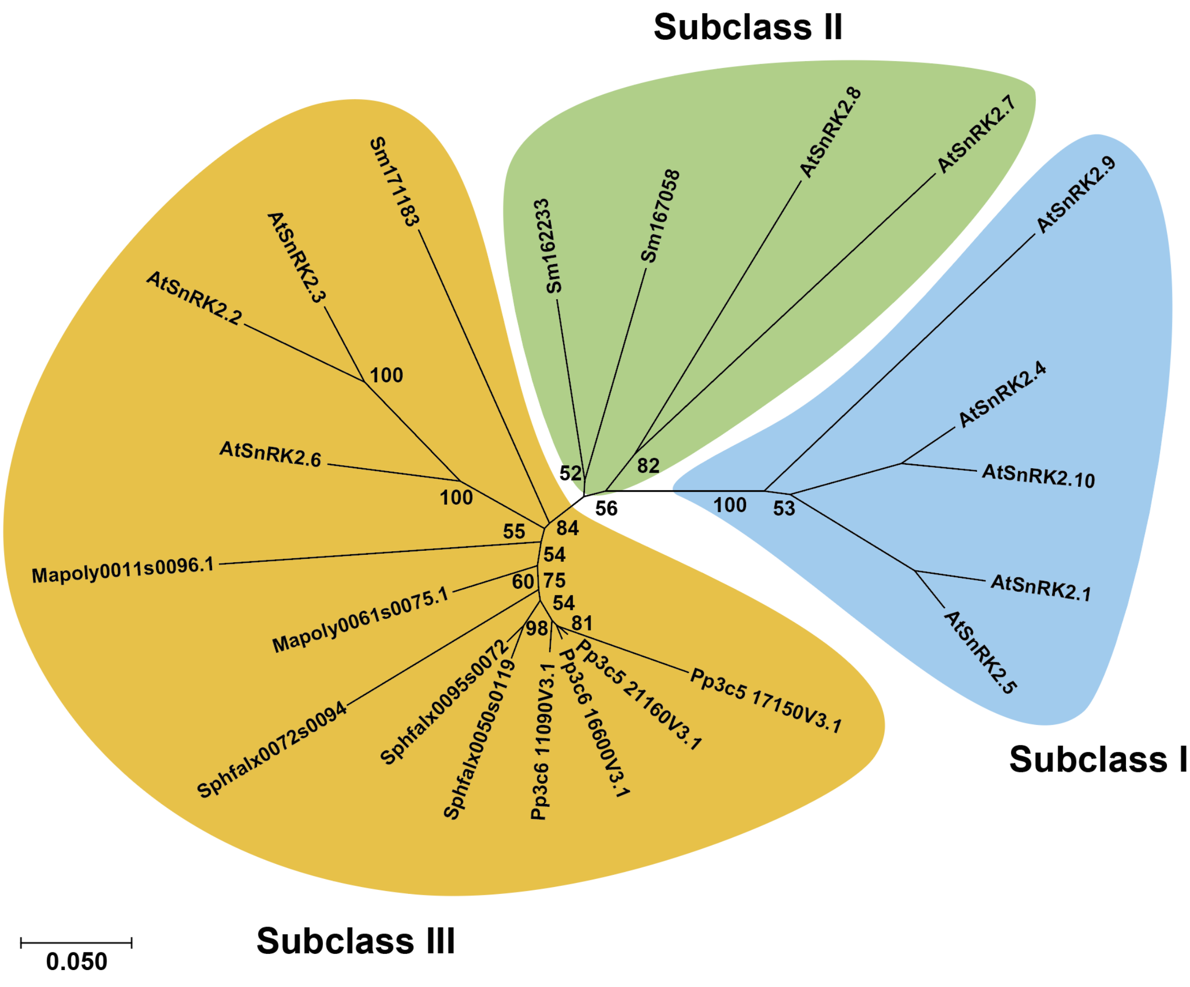

Supplementary Figure 11 (Shinozawa et al.)

Supplementary Figure 11 | Phylogram of SnRK2-type protein kinases from the lycophyte *Selaginella moellendorffii* and bryophytes.

Amino acid sequences from *Marchantia polymorpha* (Mapoly), *Sphagnum fallax* (Sphfalx), *Physcomitrella patens* (Pp), *Selaginella moellendorffii* (Sm) and *Arabidopsis thaliana* (At) were used for phylogenetic analysis (data source is shown in Supplementary Data 9). The sequences were aligned with ClustalW. A neighbor-joining tree was constructed based on alignment of the amino acid sequences containing the kinase and regulatory domains. The phylogram shown is unrooted. Numbers on branches indicate bootstrap values (> 50 %) from 1,000 replicates. The bar represents number of amino acid changes per branch length.

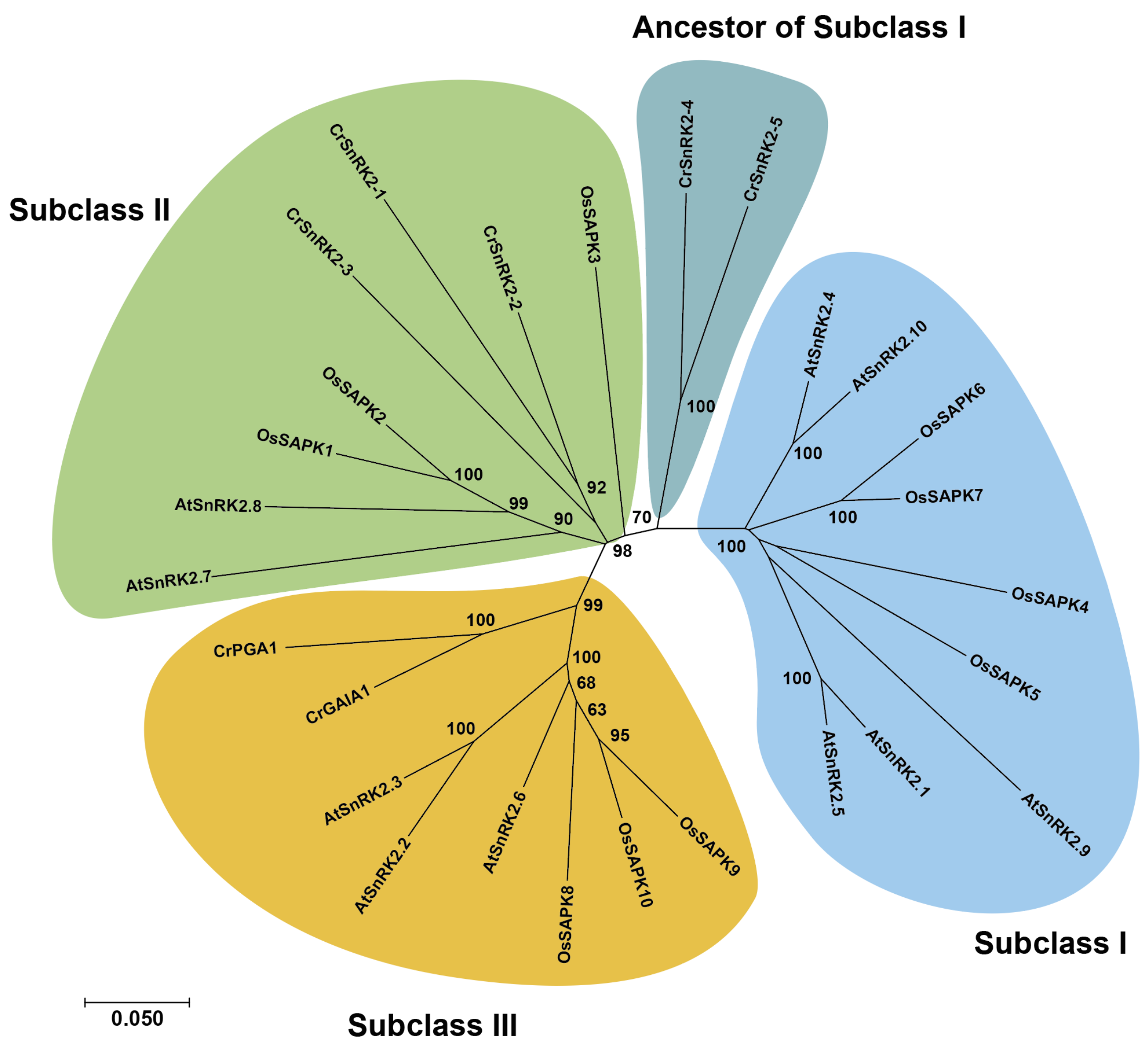

Supplementary Figure 12 (Shinozawa et al.)

Supplementary Figure 12 | Phylogram of SnRK2-type protein kinases from ferns and spermatophytes.

Amino acid sequences from *Ceratopteris richardii* (Cr), *Oryza sativa* (Os) and *Arabidopsis thaliana* (At) were used for phylogenetic analysis (data source is shown in Supplementary Data 9). The sequences were aligned with ClustalW. A neighbor-joining tree was constructed based on alignment of the amino acid sequences containing the kinase and regulatory domains. The phylogram shown is unrooted. Numbers on branches indicate bootstrap values (> 50 %) from 1,000 replicates. The bar represents number of amino acid changes per branch length.

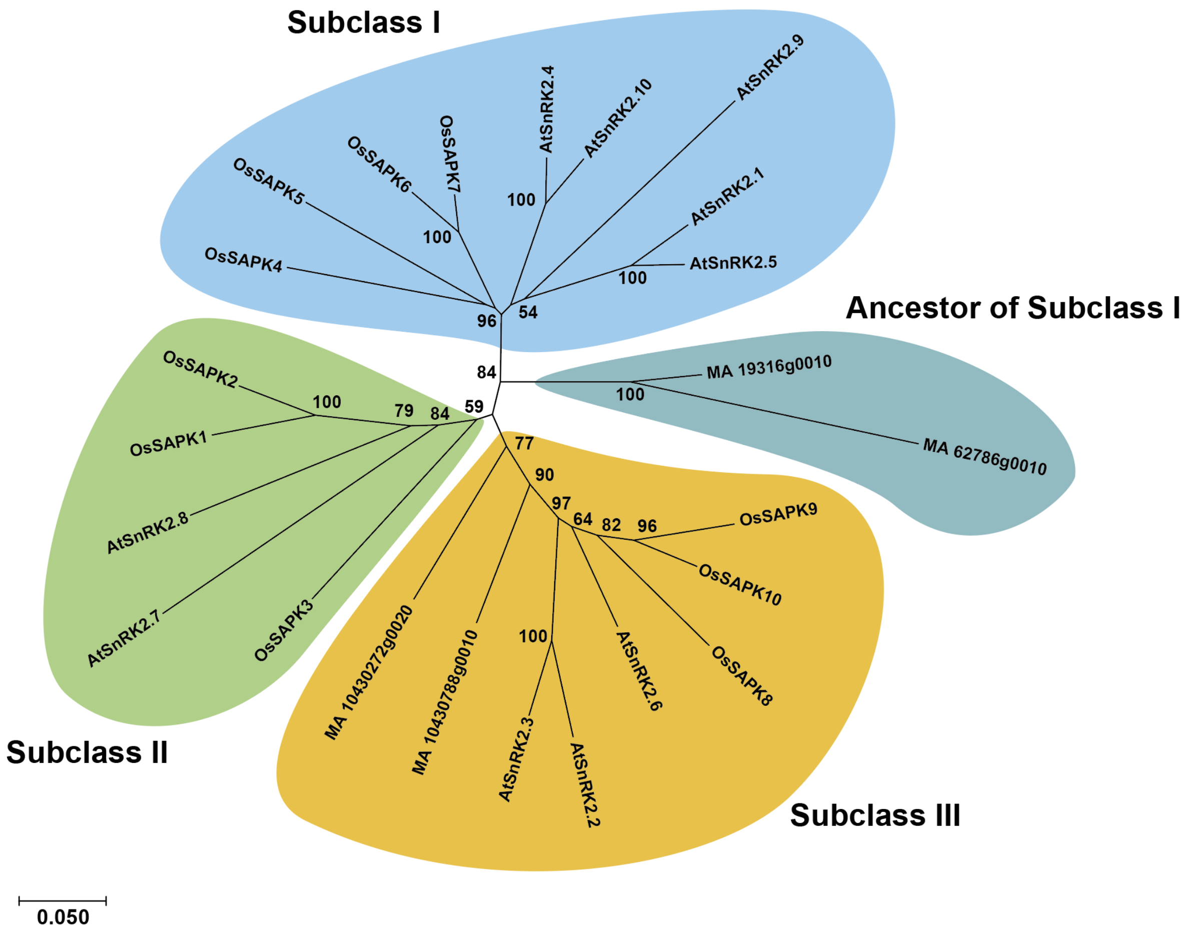

Supplementary Figure 13 (Shinozawa et al.)

Supplementary Figure 13 | Phylogram of SnRK2-type protein kinases from gymnosperms and spermatophytes.

Amino acid sequences from *Picea abies* (MA), *Oryza sativa* (Os) and *Arabidopsis thaliana* (At) were used for phylogenetic analysis (data source is shown in Supplementary Data 9). The sequences were aligned with ClustalW. A neighbor-joining tree was constructed based on alignment of the amino acid sequences containing the kinase and regulatory domains. The phylogram shown is unrooted. Numbers on branches indicate bootstrap values (> 50 %) from 1,000 replicates. The bar represents number of amino acid changes per branch length.

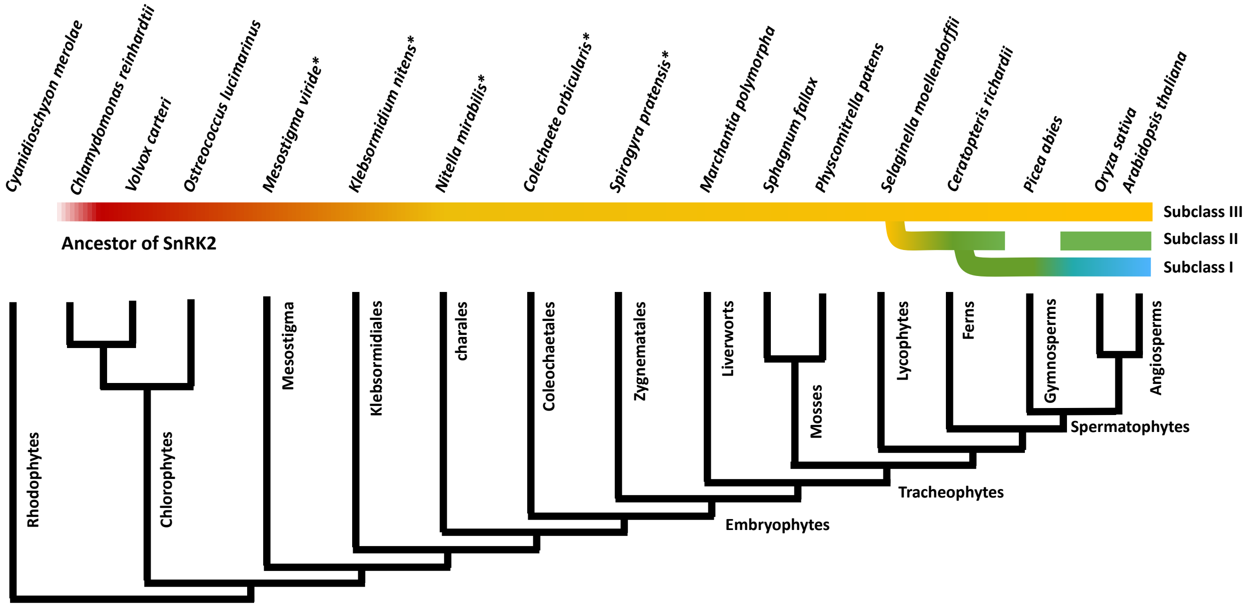

Supplementary Figure 14 (Shinozawa et al.)

Supplementary Figure 14 | Evolutionary model of SnRK2-type protein kinases.

The model was drawn based on phylogenetic analysis shown in Supplementary Figures 10-13. Species name marked with asterisk indicates that gene models were supported only by transcriptome data.

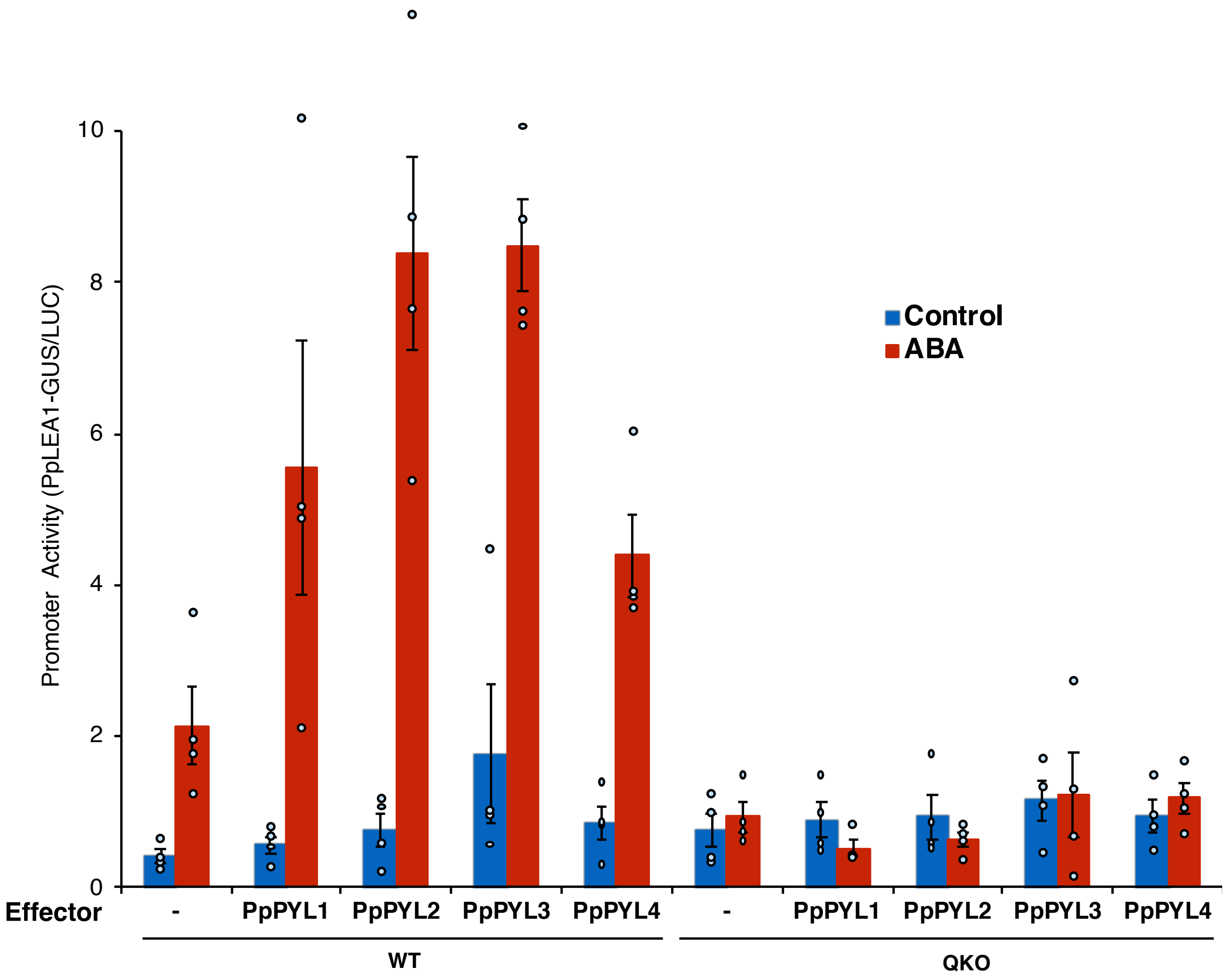

Supplementary Figure 15 (Shinozawa et al.)

Supplementary Figure 15 | Evaluation of PpPYLs in ABA signaling in *P. patens*.

PpPYL genes (*PpPYL1: Pp3c26_15240*, *PpPYL2: Pp3c13_7110*, *PpPYL3: Pp3c7_26290*, and *PpPYL4: Pp3c9_19760*) were fused to the rice *actin* promoter and introduced into WT or QKO protonemata with *PpLEA1-GUS* and *Ubi-LUC* constructs and treated with or without 10 µM ABA for 1d. Levels of gene expression are represented by GUS per LUC ratio. Error bars indicate the SE (n = 4).

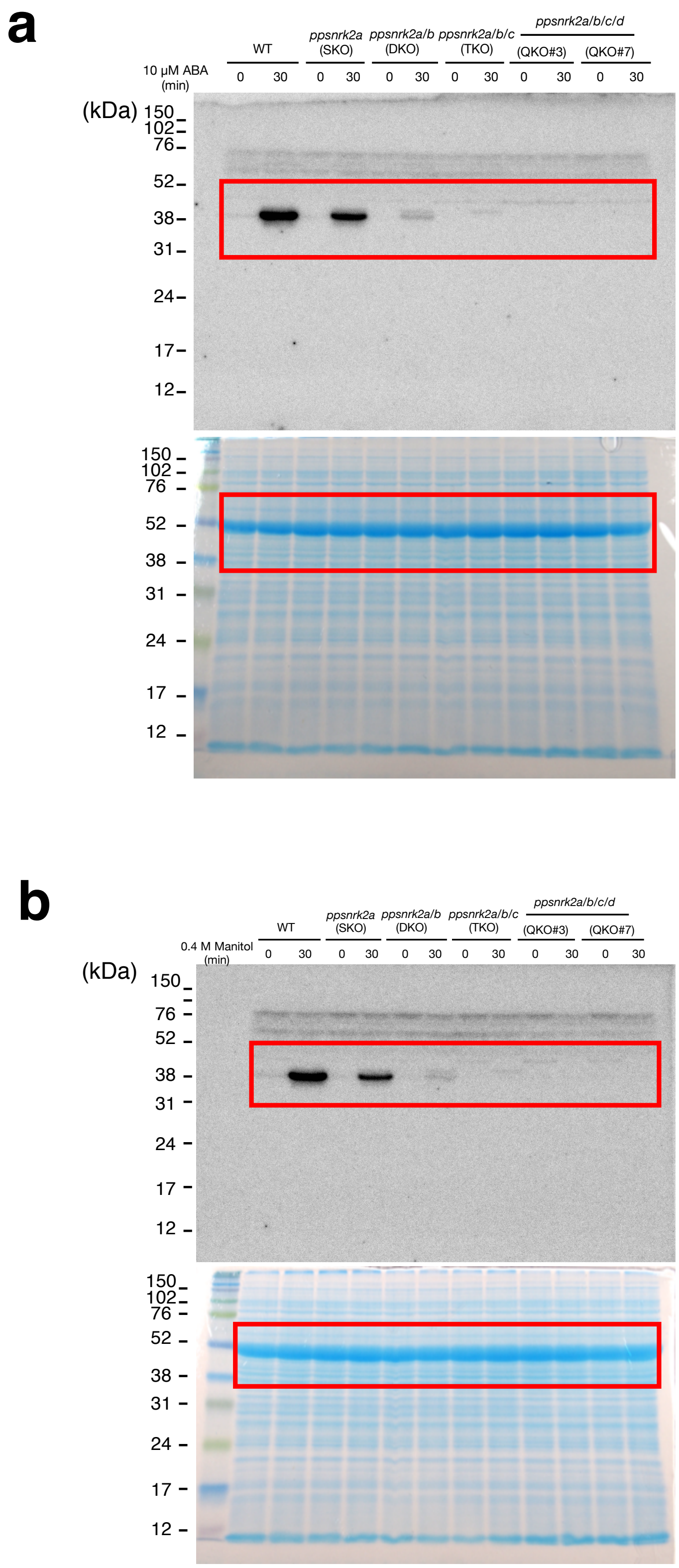

Supplementary Figure 16 (Shinozawa et al.)

Supplementary Figure 16 | Full-length blot images used in Figure 1a,b.

The panels used are boxed. The molecular weights (kDa) of the marker proteins are indicated on the left of the images.

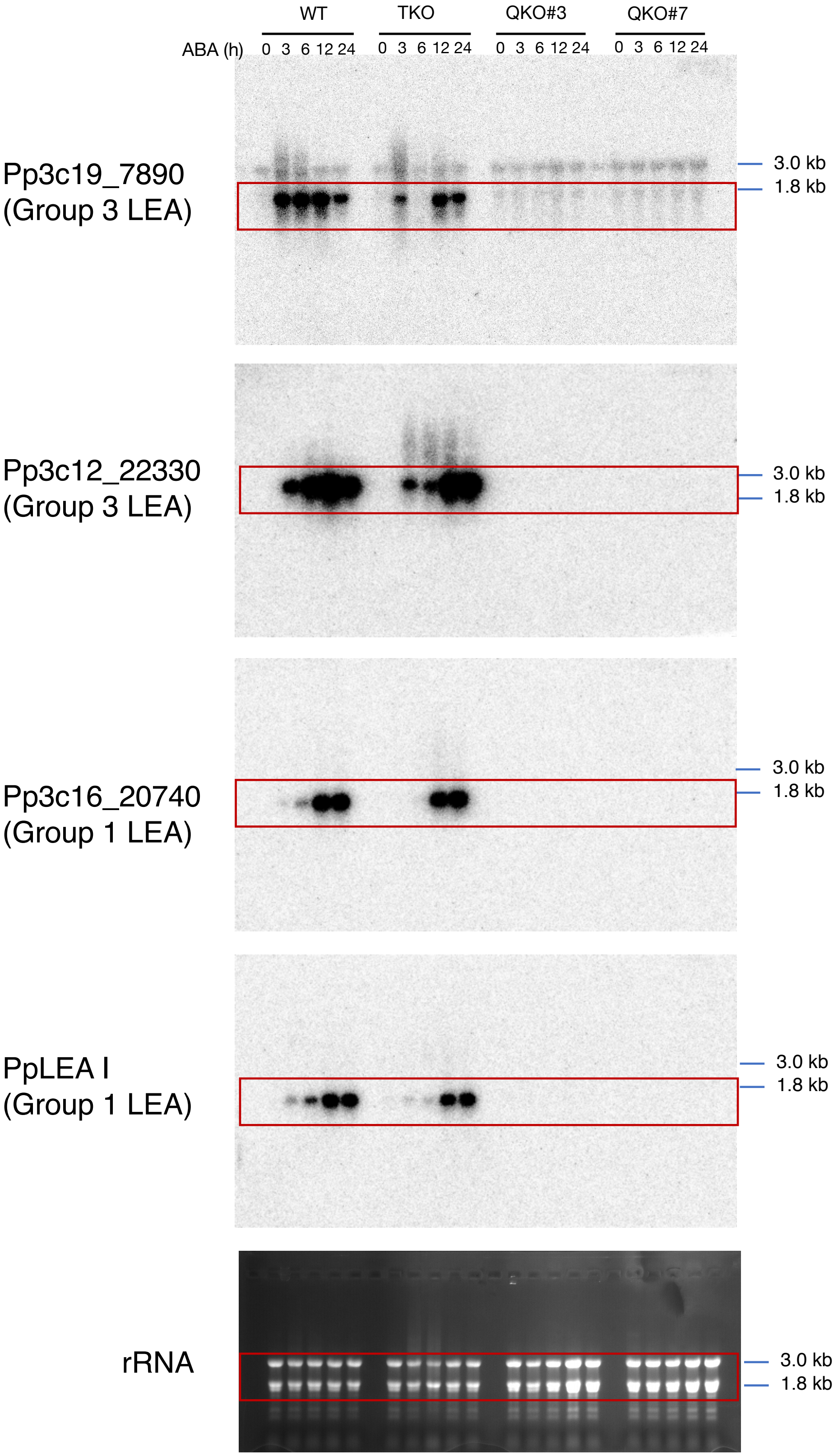

Supplementary Figure 17 (Shinozawa et al.)

Supplementary Figure 17 | Full-length blot images used in Figure 1d.

The panels used are boxed. The molecular sizes (kb) of 26S rRNA and 18S rRNA are indicated on the right of the images.

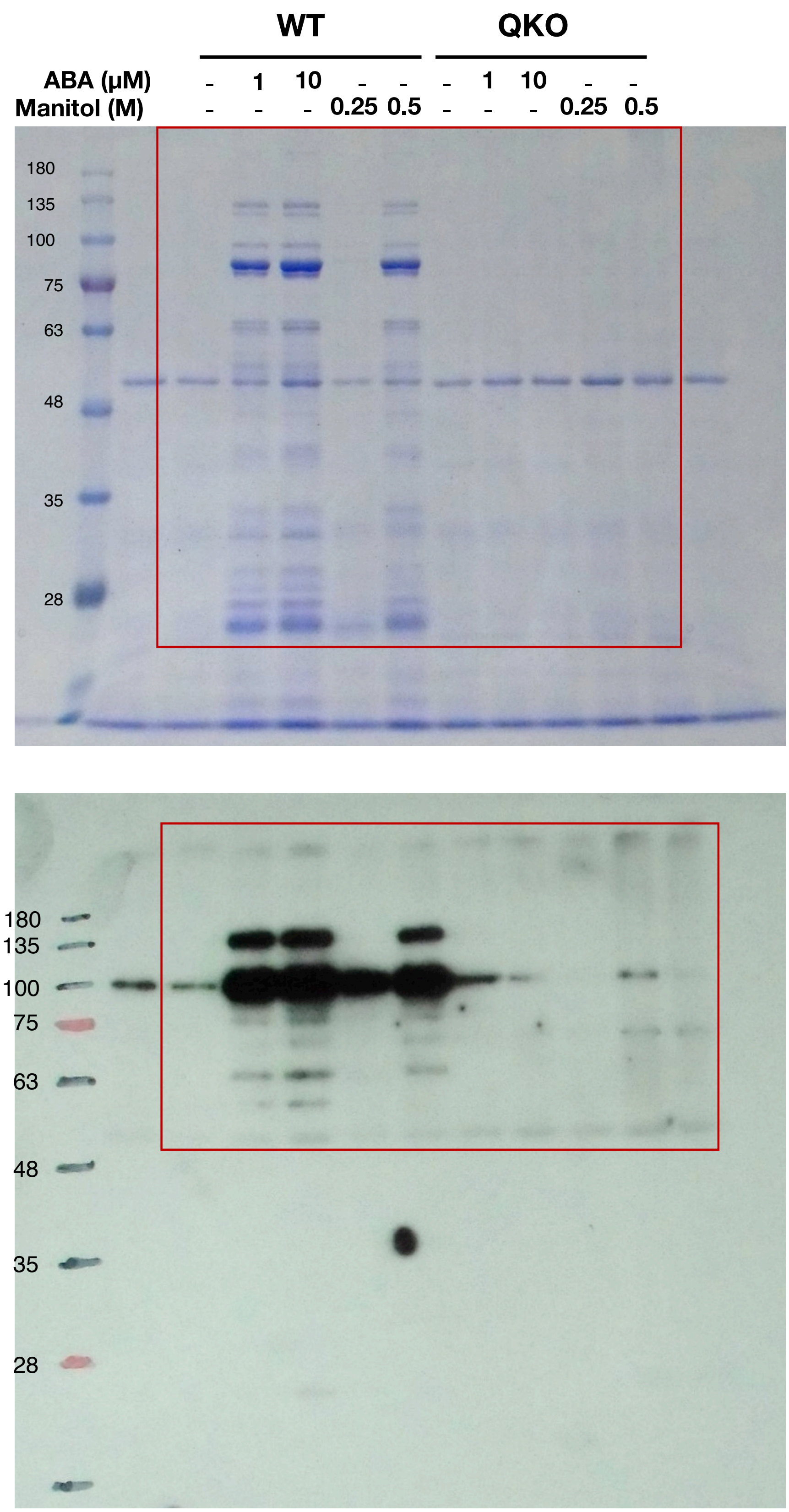

Supplementary Figure 18 (Shinozawa et al.)

Supplementary Figure 18 | Full-length blot images used in Figure 1e,f.

The panels used are boxed. The molecular weights (kDa) of the marker proteins are indicated on the left of the images.

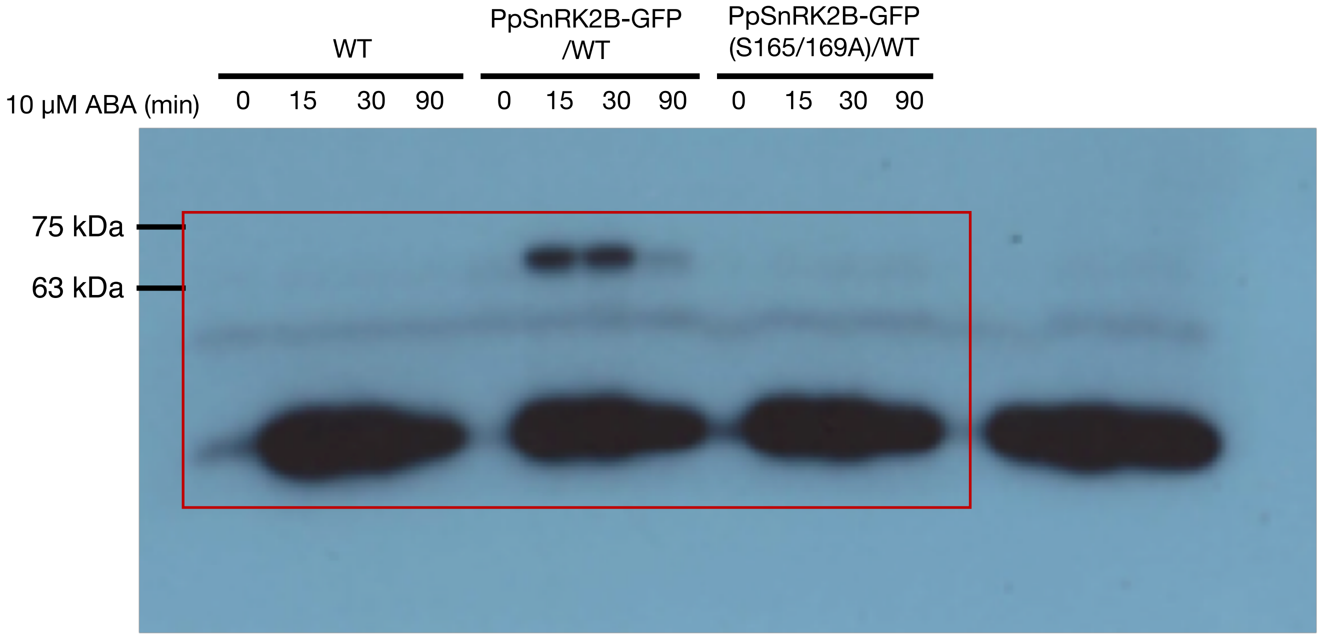

Supplementary Figure 19 (Shinozawa et al.)

Supplementary Figure 19 | Full-length blot image used in Figure 4a.

The panels used are boxed. The molecular weights (kDa) of the marker proteins are indicated on the left of the images.

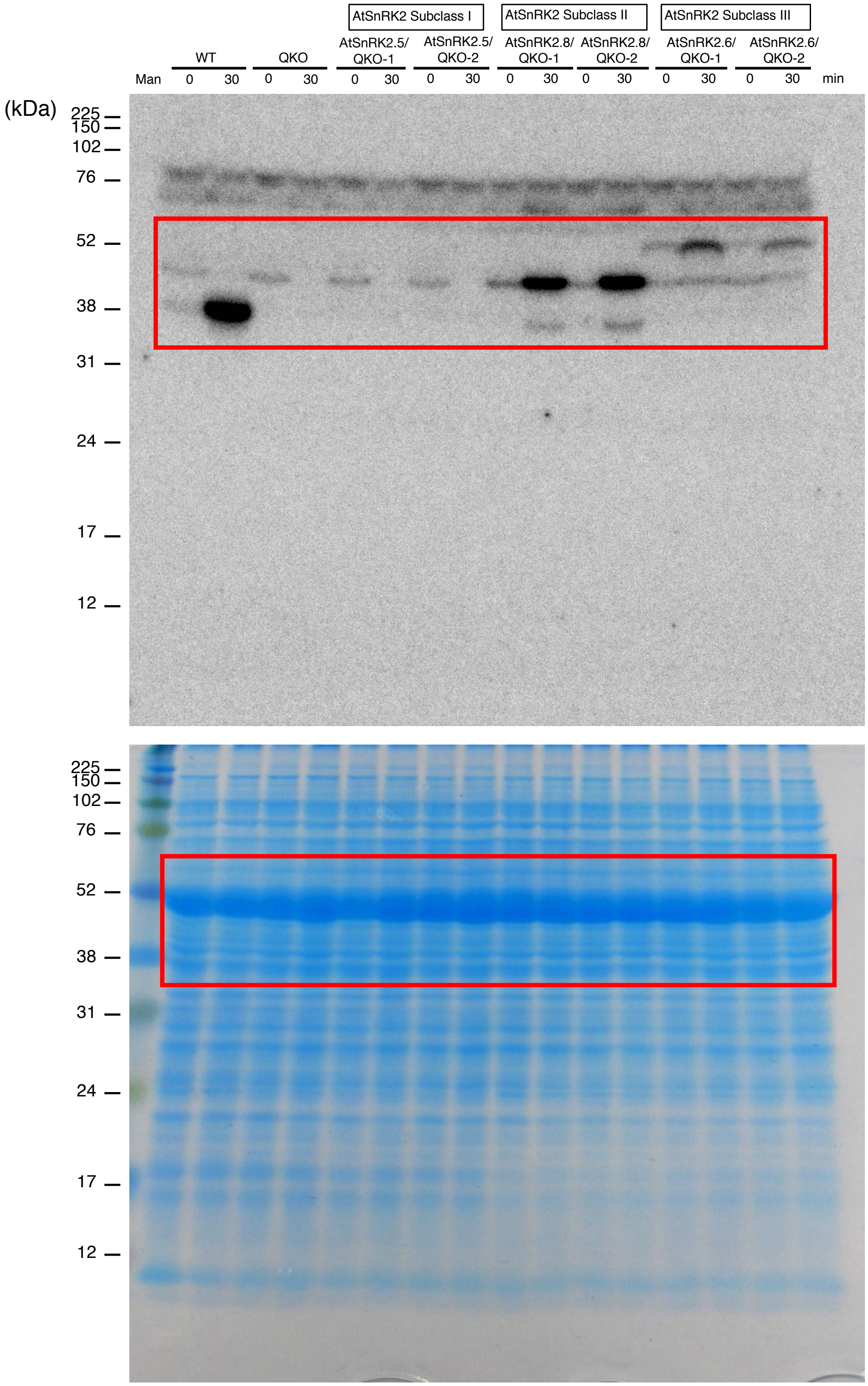

Supplementary Figure 20 (Shinozawa et al.)

Supplementary Figure 20 | Full-length blot image used in Figure 5c.

The panels used are boxed. The molecular weights (kDa) of the marker proteins are indicated on the left of the images.

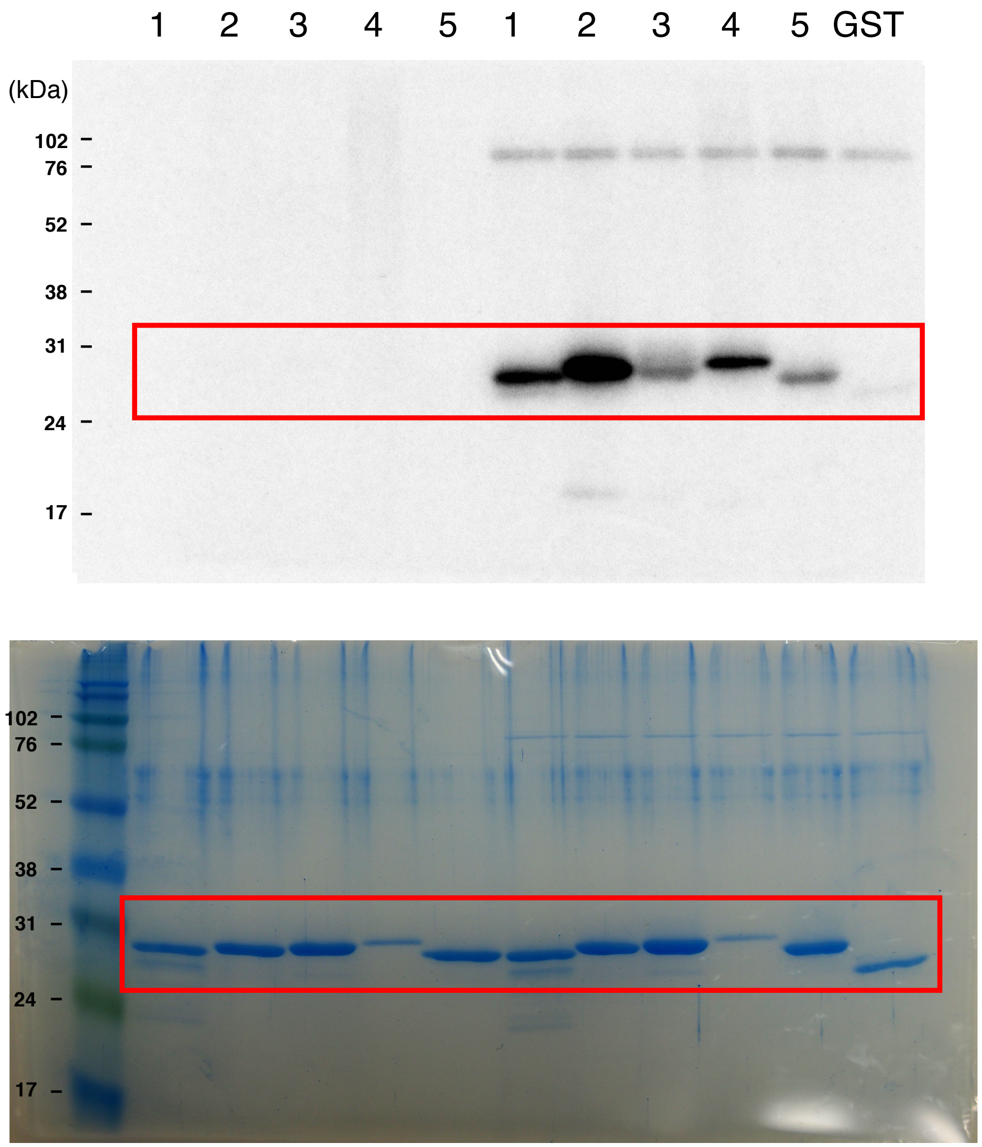

Supplementary Figure 21 (Shinozawa et al.)

Supplementary Figure 21 | Full-length blot images used in Supplementary Figure 6.

The panels used are boxed. The molecular weights (kDa) of the marker proteins are indicated on the left of the images.

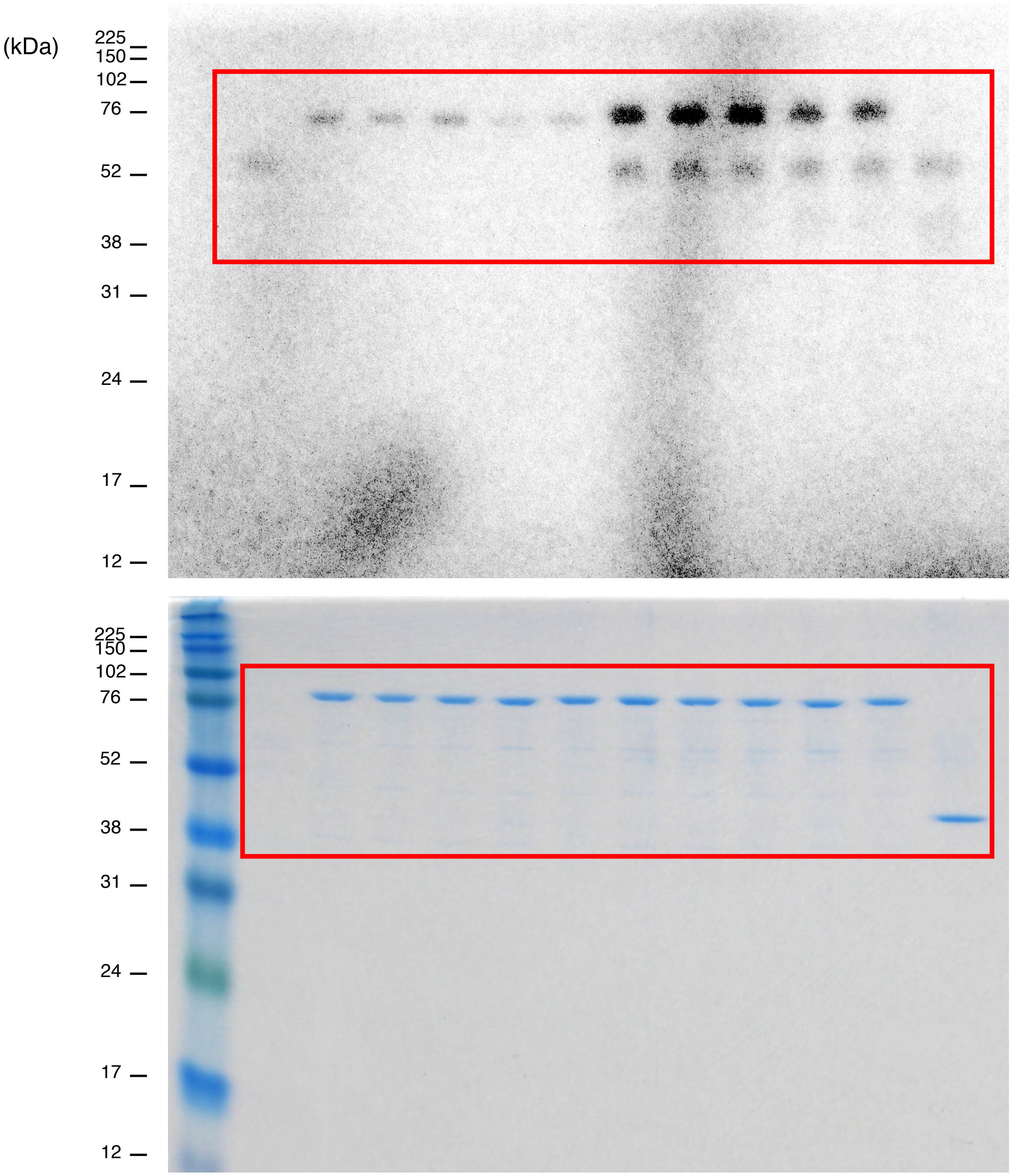

Supplementary Figure 22 (Shinozawa et al.)

Supplementary Figure 22 | Full-length blot image used in Supplementary Figure 7c.
The panels used are boxed. The molecular weights (kDa) of the marker proteins are indicated on the left of the images.

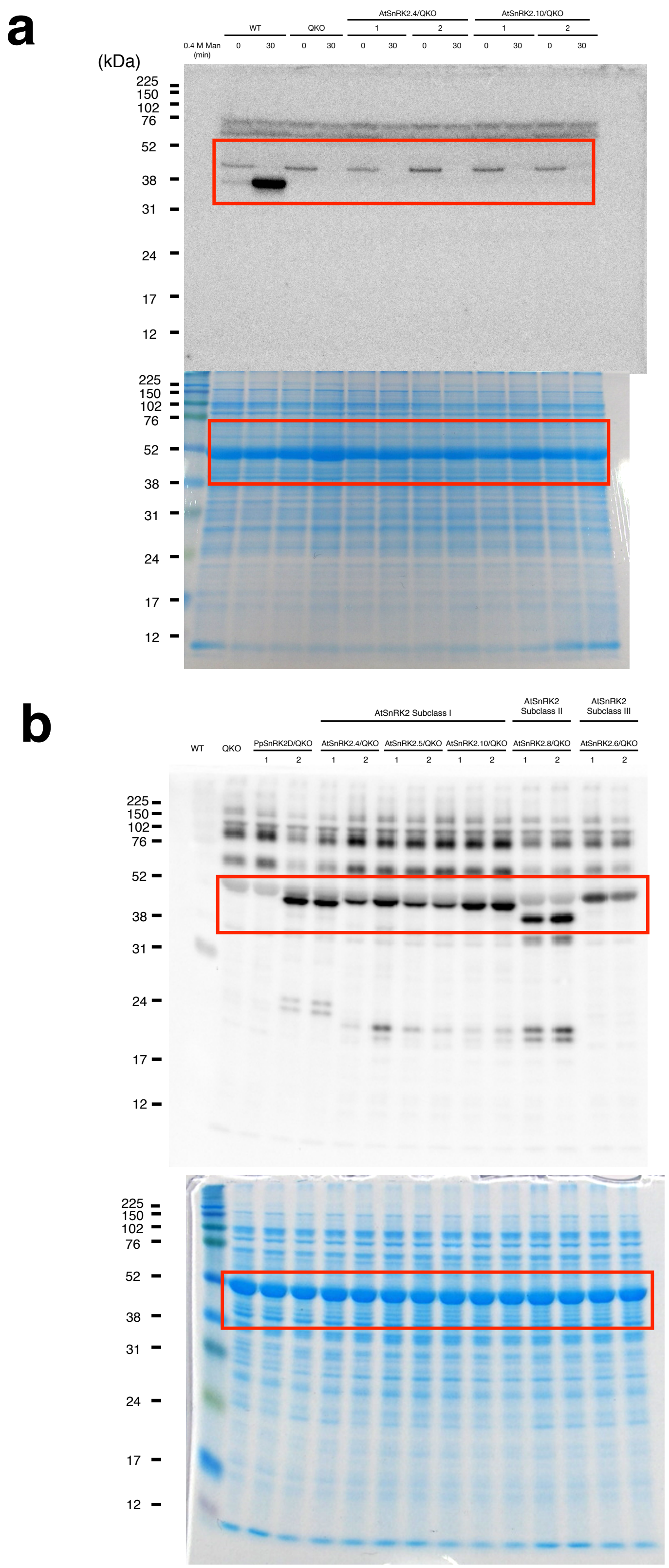

Supplementary Figure 23 (Shinozawa et al.)

Supplementary Figure 23 | Full-length blot image used in Supplementary Figure 8b, c.

The panels used in Supplementary Figure 8b (**a**) and Supplementary Figure 8c (**b**) are boxed. The molecular weights (kDa) of the marker proteins are indicated on the left of the images.
